# Covalent and Non-covalent In-Flow Biofunctionalization for Capture Assays on Silicon Chips: White Light Reflectance Spectroscopy Immunosensor Combined with TOF-SIMS Resolves Immobilization Stability and Binding Stoichiometry

**DOI:** 10.1021/acs.langmuir.3c01181

**Published:** 2023-07-12

**Authors:** Katarzyna Gajos, Alicja Orzech, Karolina Sanocka, Panagiota Petrou, Andrzej Budkowski

**Affiliations:** † M. Smoluchowski Institute of Physics, Jagiellonian University, Łojasiewicza 11, Kraków 30-348, Poland; ‡ Institute of Nuclear & Radiological Sciences & Technology, Energy & Safety, NCSR Demokritos, P. Grigoriou & Neapoleos Street, Aghia Paraskevi, Athens 15341, Greece

## Abstract

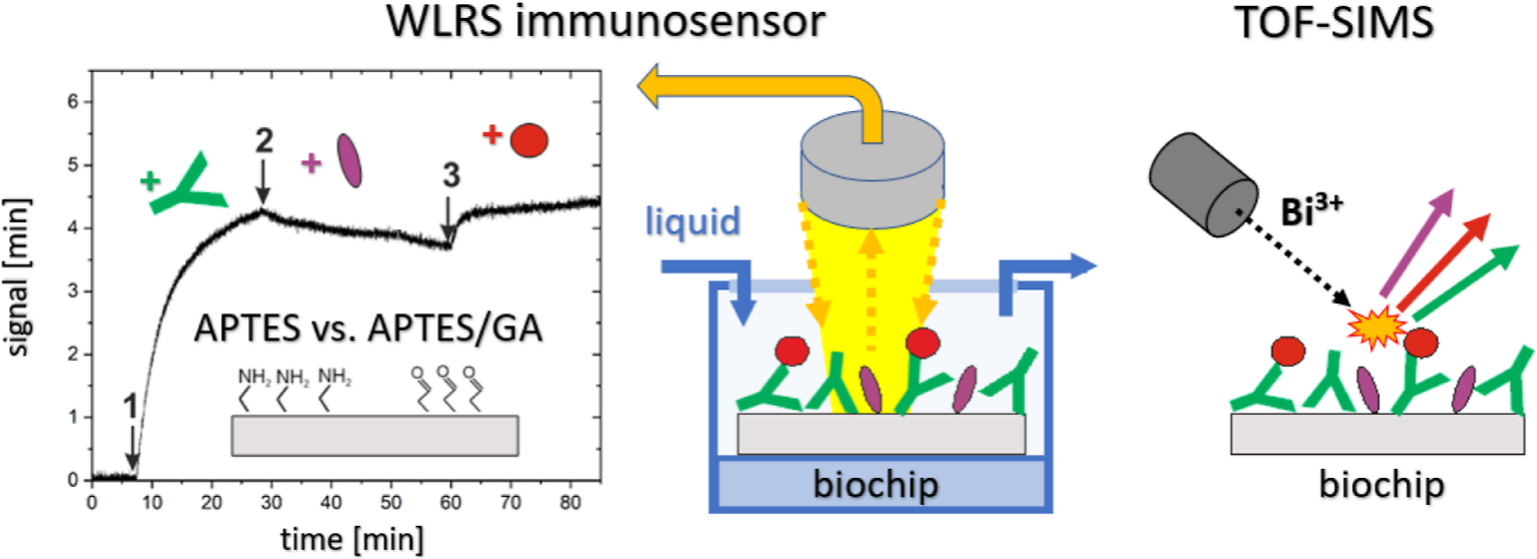

Immunosensors that combine planar transducers with microfluidics to achieve in-flow biofunctionalization and assay were analyzed here regarding surface binding capacity, immobilization stability, binding stoichiometry, and amount and orientation of surface-bound IgG antibodies. Two IgG immobilization schemes, by physical adsorption [3-aminopropyltriethoxysilane (APTES)] and glutaraldehyde covalent coupling (APTES/GA), followed by blocking with bovine serum albumin (BSA) and streptavidin (STR) capture, are monitored with white light reflectance spectroscopy (WLRS) sensors as thickness *d*_Γ_ of the adlayer formed on top of aminosilanized silicon chips. Multi-protein surface composition (IgG, BSA, and STR) is determined by time of flight secondary ion mass spectrometry (TOF-SIMS) combined with principal component analysis (applying barycentric coordinates to the score plot). In-flow immobilization shows at least 1.7 times higher surface binding capacity than static adsorption. In contrast to physical immobilization, which is unstable during blocking with BSA, chemisorbed antibodies desorb (reducing *d*_Γ_) only when the bilayer is formed. Also, TOF-SIMS data show that IgG molecules are partially exchanged with BSA on APTES but not on APTES/GA modified chips. This is confirmed by the WLRS data that show different binding stoichiometry between the two immobilization schemes for the direct binding IgG/anti-IgG assay. The identical binding stoichiometry for STR capture results from partial replacement with BSA of vertically aligned antibodies on APTES, with fraction of exposed Fab domains higher than on APTES/GA.

## 1 Introduction

*Biofunctionalization* of the surface of the biosensor that involves the immobilization of capture molecules is a crucial issue for the development of biosensors that are increasingly being used nowadays in medical diagnostics, food safety monitoring, and pollution detection.^1^ The performance of biosensors is determined by the quality of the functional biomolecular layer on the sensor/liquid interface in terms of the surface density of the capture molecules, the *stability* of *immobilization*, and the efficiency of analyte capture.^1^ In the case of immunosensors, immobilization of antibodies, which act as capture molecules, requires special attention due to the antigen binding efficiency that depends on the *orientation* adopted by surface immobilized antibodies.^2−4^ Although the immobilization scheme controls the molecular orientation,^2−4^ the latter is also determined by surface density, with a more vertical arrangement forced by a decreasing surface area accessible to each molecule.^5^ Recently, antibody immobilization described by random sequential adsorption,^6^ forming random rather than close-packed molecular arrangement, was shown to describe the relationship between antibody orientation and its surface density.^7,8^ The stability of the immobilization of the capture protein could be hindered by partial *desorption* or *exchange* with other molecules that may occur during subsequent steps of biofunctionalization and assay. The observation of these complex phenomena, which involve protein–surface and protein–protein interactions,^9,10^ is difficult due to the question of protein composition in formed multi-molecular adlayers.^11−13^ For biofunctionalization and assay protocols, molecular desorption and exchange have been reported mainly for immobilization based on the physical adsorption approach,^14−16^ however, they can also occur for covalent immobilization schemes under certain conditions.^7^ An undetected reduction in the surface amount of capture or blocking molecules could lead to misinterpretation of the biosensor signal. In particular, the partial exchange of proteins already immobilized with other molecules that are introduced onto the surface is not resolved by the response of biosensors sensitive to the cumulative mass of all molecules. As a result, the biosensor response to an assay could lead to an inaccurate value of the *binding stoichiometry*. Surface analysis methods,^2,3^ such as spectroscopic ellipsometry (SE), white light reflectance spectrometry (WLRS), quartz crystal microbalance, surface plasmon resonance, X-ray photoelectron spectroscopy^14,17^ and dual polarization interferometry,^18,19^ also reveal the cumulative surface density of all biomolecules. Therefore, to address this issue, a comprehensive analysis of the biomolecular layers at the biosensor surface should involve *molecular discrimination*. In this case, molecule labeling methods, i.e., fluorescence, radiolabeling, and colorimetric detection, could be applied; however, they require molecule engineering, which cannot be performed for all molecules involved in functionalization and assay protocols. In contrast, time of flight secondary ion mass spectrometry (TOF-SIMS) is a powerful surface sensitive technique, which offers a label-free examination of multi-molecular layers and opens recently reviewed perspectives for biosensor surface analysis.^17^ A comparative analysis of the layer content of each individual protein involved in biosensor functionalization and assay is possible on the basis of differences in its amino acid composition.^14,16,17^

Regarding the approaches applied for capture protein immobilization onto the sensor surface, the most widely used are based on physical adsorption, covalent coupling, or affinity binding.^1^*Covalent attachment* of the protein probe is often preferred because of the expected stability of immobilized molecules under flow conditions, which allows the biosensor to regenerate and reuse its functionalized surface. However, also for *physical adsorption* strategies, which are easily applied due to their simplicity and repeatability, stable biosensor performance with regeneration possibility has been reported.^20^ Immobilization of biomolecules generally requires modification of the biosensor surface to provide the chemical properties that promote physical adsorption or allow covalent coupling.^1,21^ For this purpose, the application of self-assembled monolayers involving alkanethiols and alkoxysilanes is a robust and versatile approach for modification of gold and silicon-based surfaces, respectively.^21,22^ For silicon-based biosensors, surface modification with amino-terminated silane, 3-aminopropyltriethoxysilane (APTES), is a primary strategy for the physical adsorption of proteins, while subsequent activation of the APTES layer with glutaraldehyde is a common procedure for the covalent coupling of proteins.^1,21^ Although glutaraldehyde activation is considered to prevent protein desorption, the impact of this procedure on the stability of the immobilization has not been explicitly examined. Previous comparative analyses between physical adsorption and covalent attachment have focused their effect in assay efficiency^23,24^ or antibody orientation.^8^

The deposition of biomolecules on the sensor surface from solution could be realized by the *in-flow* strategy or under static conditions involving immersion in solution or bioprinting techniques. Despite the fact that in most biosensors a microfluidic module is typically involved to allow flow of the sample solution and real-time monitoring of the layer formation during the *assay*, immobilization of the capture molecules is achieved frequently under static conditions before assembling the microfluidic module^25^ rather than using an in situ in-flow strategy. The advantages of the latter are the opportunity to monitor the formation of the capture molecule layer and also the spatial limitation of their immobilization to the array covered by the fluidic channels. The in-flow approach has already been applied for the *biofunctionalization* of nanophotonic interferometric biosensors^26−28^ and SERS based biosensor capillary networks,^29^ as well as for the immobilization of enzymes within microfluidic channels for flow reactor systems.^30,31^ The protein layer formation process could differ for static and in-flow immobilization strategies due to additional hydrodynamic shear forces that appear under flow conditions.^32^

In this work, we report a complete comparative examination of the *in-flow biofuctionalization* and *assay* on aminosilanized silicon chips involving the physical adsorption (APTES) or glutaraldehyde coupling (APTES/GA) of IgG antibodies. This extends our previous studies that examined biosensor interface functionalization protocols that involve immobilization of molecules under static conditions reviewed in ref (17). Here, physical adsorption and covalent coupling biofunctionalization strategies are compared in terms of *surface binding capacity*, immobilization stability, and binding stoichiometry for capture and direct assay formats. Both immobilization schemes resulted in the surfaces with an adjusted amount of antibody adjusted from low coverage to second layer formation, consistent with the random packed molecular arrangement described recently for the IgG monolayers on the APTES and APTES/GA modified silicon.^7,8^ Real-time monitoring of the thickness of the biomolecular layer with a model optical sensor based on WLRS^20^ is juxtaposed with *multi-protein composition* analysis with ToF-SIMS supported by multivariate principal component analysis (PCA), which is extended here by barycentric coordinates applied to the score plot. In addition, capture and direct assay formats are compared to contrast the surface-bound IgG molecules that act as antibodies and antigens. This complementary examination provides discrimination between different molecules (antibodies, blocking molecules, and antigens) and enables insight into surface phenomena that determine the *immobilization stability* and *binding stoichiometry*. In particular, we examine the *desorption* and *exchange* of IgG molecules in the course of biofunctionalization and assay procedures and evaluate their extents depending on the immobilization strategy and the surface amount of the antibodies. We show that the appearance of these phenomena also depends on the *orientation* of the antibody, which varies with its surface density.^7,8^ Moreover, we determine how the binding stoichiometry of the capture assay observed with the biosensor response is defined by both the immobilization stability and the dominant antibody orientation.

## 2 Experimental Section

### 2.1 Functionalization of Silicon Substrates

Silicon substrates with a native SiO_2_ layer were purchased from Si-Mat (GmbH, Germany), while silicon chips with a 1000 nm thick SiO_2_ layer used for WLRS are from ThetaMetrisis S.A. (Athens, Greece). Before silanization, silicon substrates and chips were cleaned by sonication in toluene (POCH, Gliwice, Poland) for 10 min followed by cleaning and hydrophilization by treatment with oxygen plasma for 30 s. Modification with APTES (APTES, Sigma-Aldrich, Darmstadt, Germany) was carried out by immersion of substrates and chips in a 1% (v/v) APTES solution in toluene for 10 min. The samples were then subsequently sonicated in toluene and ethanol for 10 min, dried under a nitrogen stream, and baked for 20 min at 120 °C.^7,8^ Surface activation with aldehyde groups for APTES/GA samples was performed by immersion of the APTES modified substrates and chips in a 2.5% (v/v) aqueous glutaraldehyde solution (Sigma-Aldrich, Darmstadt, Germany) for 20 min. The samples were then washed with distilled water and dried under a stream of nitrogen.

Single protein layers, used as reference samples in TOF-SIMS analysis, were prepared on silicon substrates modified with APTES and APTES/GA by static adsorption approach. For this purpose, the functionalized substrates were incubated with a 100 μL droplet of a 500 μg/mL protein solution in phosphate buffered saline (PBS) buffer (pH 7.4, 0.15 M, Sigma-Aldrich, Darmstadt, Germany) for 30 min. Polyclonal goat anti-streptavidin (STR) antibody (anti-STR IgG) (USBiological, Salem, MA, USA), bovine serum albumin (BSA) (ACROS Organics, Geel, Beligum), and recombinant STR (Sigma-Aldrich, Darmstadt, Germany) were used. After incubation with protein solution, silicon substrates and chips were washed with buffer, distilled water, and dried under a nitrogen stream.

### 2.2 WLRS Instrumentation

WLRS measurements were performed using an FR-pRo system from ThetaMetrisis S.A. (Athens, Greece) combined with a liquid cell (FR-Microfluidic kit). The detection system consists of a broad-band UV–vis 250–700 nm light source (ThetaMetrisis S.A., Athens, Greece), a PC-controlled spectrometer (Ocean Optics Maya2000 pRo, Orleando, FL, USA), and a reflection probe composed on seven optical fibers (Ocean Optics, Orleando, FL, USA). Light emitted from the light source is directed vertically to the chip surface by six fibers arranged at the circumference of the reflection probe. The reflected light from the chip is collected and guided to the spectrometer by the seventh central fiber to be continuously recorded and analyzed.^25^ For in-flow protein immobilization and assay, transparent microfluidic modules (ThetaMetrisis S.A., Athens, Greece) were placed on top of functionalized Si/SiO_2_ chips.^20,25^ A single chip assembled with its microfluidic cell was then placed in the WLRS instrument docking station. The reflection probe was kept at a constant distance of about 3 mm from the top of the microfluidic cell for all measurements. Continuous fluid flow was achieved using a microfluidic syringe pump with flow rate control. FR-Monitor software (ThetaMetrisis S.A., Athens, Greece) was used for recording and analysis of the reflected spectra. Spectra were recorded with a 15 ms integration time and fitted in the spectral range 400–560 nm using the Levenberg–Marquardt algorithm considering the refractive indices *n*(λ) of all layers of a multilayer stack of water/protein and silane layer/SiO_2_/silicon substrates. The algorithm was applied to evaluate the initial thickness of the SiO_2_/APTES adlayer and to transform in real time the spectral shift in effective biomolecular layer thickness *d*_Γ_.^25^

### 2.3 In-Flow Biofunctionalization and Assay

First, each chip functionalized with APTES or APTES/GA was assembled with the microfluidic module, placed on the WLRS instrument docking station and equilibrated with PBS buffer (pH 7.4, 0.15 M) to acquire a stable baseline. Then, on the basis of recorded spectra, the initial thickness of the SiO_2_/APTES or SiO_2_/APTES/GA adlayer was determined. After that, the spectral shift was recorded continuously and transformed in real time to the effective biomolecular layer thickness *d*_Γ_ expressed in nanometers. For the in-flow immobilization of antibodies, the solution of rabbit gamma-globulins purified from pooled normal rabbit serum (rabbit IgG) or polyclonal goat anti-STR antibody (anti-STR IgG) was passed over the chip for 20 min with a flow rate of 20 μL/min followed by rinsing with PBS buffer. For the assay after 5 min of rinsing with PBS, a BSA solution with a concentration of 2 mg/mL was run over the chip for approximately 15 min to block the free surface sites. After another 5 min of rinsing with PBS, a solution of specific binding molecules was passed over the chip. In case of rabbit IgG immobilization, a 10 μg/mL solution of polyclonal goat anti-rabbit IgG antibody (Thermo Fisher Scientific, Rockland, USA) was run for 20 min. For immobilized anti-STR IgG, a 10 μg/mL solution of STR was run for 15 min. Finally, the chips were rinsed by passing the PBS buffer and distilled water in sequence for 5 min. The flow rate throughout the assay was 20 μL/min.

### 2.4 Surface Composition Examination with TOF-SIMS and PCA

TOF-SIMS analysis of the biomolecular layer composition was performed using a TOF.SIMS 5 instrument (ION-TOF GmbH). Bi_3_^+^ clusters produced by a 30 keV liquid metal ion gun were used as primary ions. For all measurements, a current of about 0.5 pA and an ion dose density of about 10^12^ ion/cm^2^ providing static mode conditions were applied. A low-energy electron flood gun was used for charge compensation. Positive-ion high-mass resolution TOF-SIMS spectra were acquired from several non-overlapping 100 μm × 100 μm areas of each sample with a resolution of 128 × 128 points. Mass calibration was performed with H^+^, H_2_^+^, CH^+^, C_2_H_2_^+^, and C_4_H_5_^+^ peaks. The mass resolution (*m*/Δ*m*) was higher than 8000 at the C_4_H_5_^+^ peak for all spectra.

Multivariate analysis of TOF-SIMS data was performed with PCA using the PLS Toolbox (Eigenvector Research, Manson, WA) for MATLAB (MathWorks, Inc., Natick, MA). Before running PCA the intensities of selected peaks from each spectrum were normalized to sum of selected peaks and mean-centered.

## 3 Results and Discussion

**Figure 1:**
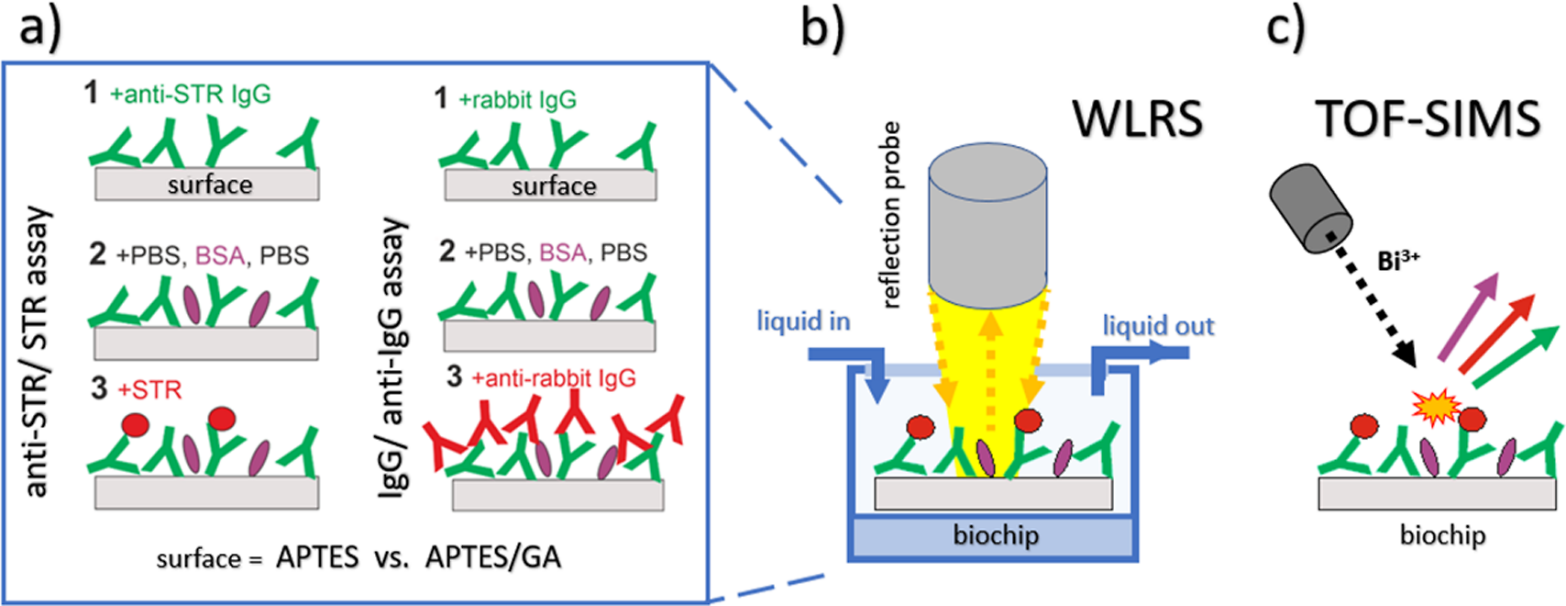
Schematic of the in-flow biofunctionalization and assay protocols carried out on a silicon chip (a), the WLRS-based biosensor monitoring the resulting changes in effective thickness of biomolecular layers (b), and TOF-SIMS analysis of the multi-protein composition of the biochip performed after protocol completion (c). Model protocols for capture and direct binding assays, with surface-immobilized IgG molecules acting as antibodies or as antigens, respectively, were examined for silicon chips modified with APTES and APTES activated with glutaraldehyde (APTES/GA). Both assays included immobilization of IgG molecules on functionalized silicon chips (1), washing and blocking of free surface sites (2), and immunoreaction (3). The anti-STR IgG/STR protocol involved the immobilization of anti-STR IgG, blocking with BSA, and STR antigen capture (capture assay), while the IgG/anti-IgG protocol involved immobilization of rabbit IgG antigens, blocking with BSA, and binding of the anti-rabbit IgG antibody (direct binding assay).

**Figure 2:**
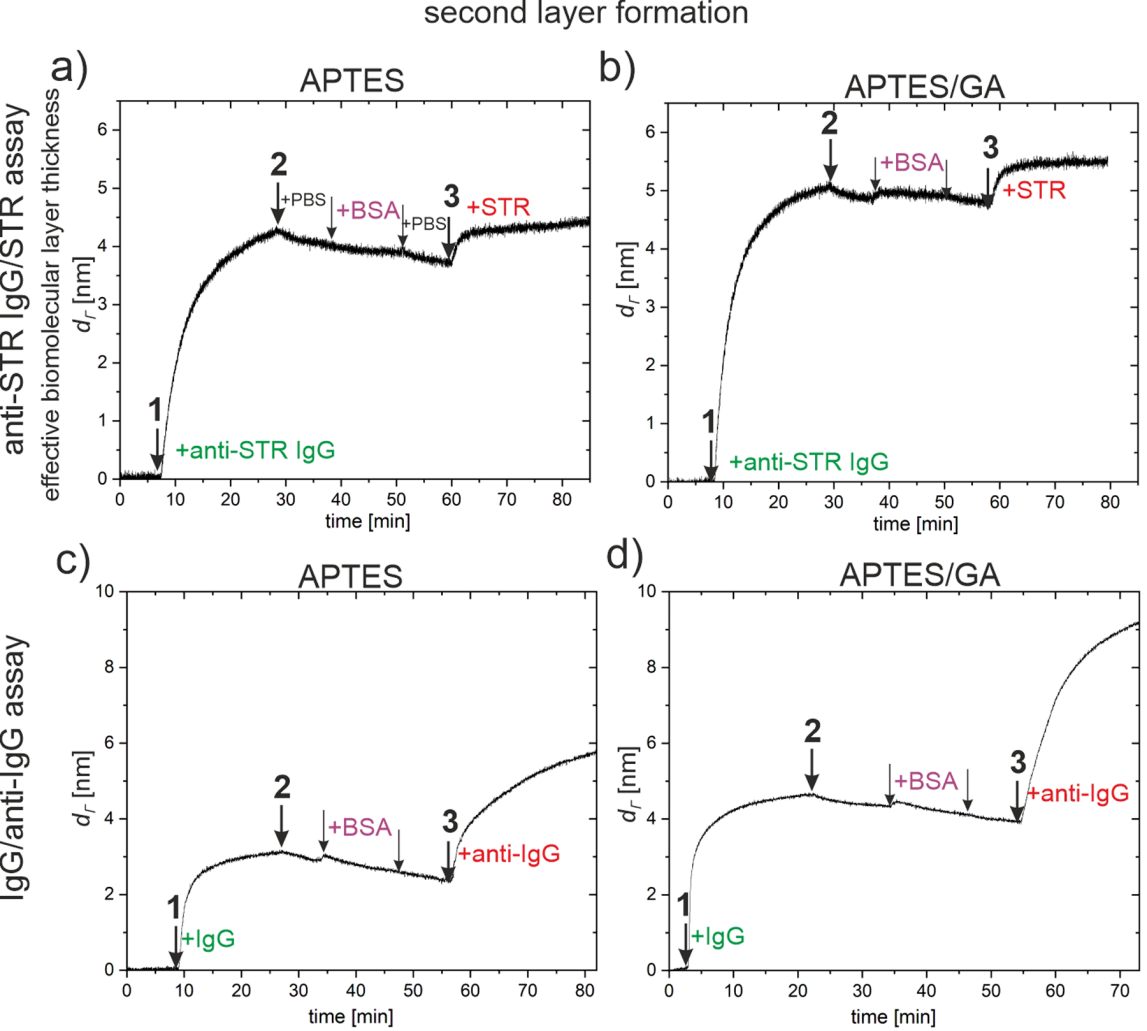
Effective thickness of the biomolecular layer *d*_Γ_ monitored in real time with the WLRS-based biosensor for the in-flow biofunctionalization and assay protocols performed on silicon chips modified with APTES (a,c) and APTES/GA (b,d), respectively. Two types of model assays were examined, anti-STR IgG/STR capture assay (a,b) and IgG/anti-IgG direct binding assay (c,d). The presented results were obtained using a high concentration (>100 μg/mL) of IgG solutions (anti-STR IgG antibody or rabbit IgG) during the immobilization step (1), resulting in the formation of a second layer of immobilized IgG molecules. The arrows indicate the start of each one of the subsequent protocol steps and mark the solution run over the biochip.

In the present work, we examine and compare in-flow strategies for biofunctionalization of amino-silane modified silicon chips with IgG molecules involving physical adsorption and glutaraldehyde covalent coupling. For a comprehensive analysis considering the immobilization capacity and stability, as well as the binding stoichiometry and dominant orientation of surface-bound IgG molecules, we applied two immunoassay configurations, which are depicted in Figure 1. In the first configuration of the capture assay, immobilized IgG molecules act as antibodies, while in the second configuration of the direct binding assay, they act as antigens. The model capture assay involved an antibody–antigen pair of goat anti-STR IgG and STR (referred to as the anti-STR/STR assay). The model direct binding assay involved the rabbit IgG - goat anti-rabbit IgG system (referred to as the IgG/anti-IgG assay). In the following sections, we present an analysis of the main steps of the assay procedure that involved immobilization of IgG molecules (1), washing with PBS buffer and blocking of free surface sites with BSA (2), and immunoreaction with a specific antigen or antibody depending on the assay configuration (3). All steps of the assay procedure were performed under constant flow conditions in the microfluidic module of the WLRS platform, as described in detail in Section 2.3. The building of the biomolecular layer in the course of the assay procedure was monitored in real time with the WLRS instrument, which transforms the shift in the reflected interference spectrum caused by the adsorption of molecules into an effective thickness *d*_Γ_ of the biomolecular layer. The WLRS instrument in such a configuration is capable of working as a biosensing platform, which has already been applied for the analysis of contaminants in drinking water and beverages (Figure 1b).^33^ For both assay configurations and immobilization methods, representative WLRS sensor responses of the effective thickness *d*_Γ_ of the biomolecular layer versus time are presented in Figures 2 and S1 in Supporting Information. They reflect situations for different surface amounts of IgG molecules, corresponding to the formation of a second layer as well as to the completion of a monolayer or a low surface coverage with the immobilized molecules. The changes in adlayer thickness are examined and discussed in the following sections after each step of the assay procedure, i.e., immobilization of IgG molecules (Section 3.2), blocking step (Section 3.3), and immunoreaction (Section 3.4). However, prior to this, the molecular composition of the adlayers resulting from the anti-STR/STR assay is evaluated with TOF-SIMS in Section 3.1 (Figure 1c).

### 3.1 Biofunctionalization and Assay Protocol: Multi-Protein Surface Composition Evaluated with Off-Flow End-of-Assay TOF-SIMS Analysis

The WLRS-based biosensor monitors the effective thickness *d*_Γ_ of the multi-component biomolecular layer, which reflects the cumulative surface density Γ of *all* different proteins on the biochip, without discrimination between molecular components. Therefore, to enable the correct interpretation of the real-time WLRS signal evolutions, determined for in-flow biofunctionalization and assay protocols, the multi-protein composition of biomolecular layers resulting from these protocols should be evaluated. In particular, the TOF-SIMS technique was applied to examine the biomolecular layers for the anti-STR IgG/STR assay configuration, with chemical specificity enabled by the differences in amino acid composition of all the proteins involved (anti-STR IgG, BSA, and STR). After completion of the anti-STR IgG/STR assay, performed with the WLRS platform (Figures 2a,b and S1), the biochip was removed from the fluidic cell, washed with distilled water, and dried in a nitrogen stream to enable TOF-SIMS examination. Thus, TOF-SIMS measurements were performed to compare the biomolecular layers resulting from the in-flow protocols executed for silicon chips functionalized with APTES (Figures 2a and S1a,c) or APTES activated with glutaraldehyde (Figures 2b and S1b,d) for three different surface densities of anti-STR IgG molecules (Figures 2a,b and S1a–d). TOF-SIMS is a surface-sensitive technique, therefore the applied TOF-SIMS setup secures the sampling of a complete protein monolayer,^17^ and provides a multi-protein *surface* composition even for a protein bilayer.^14,16^

**Figure 3:**
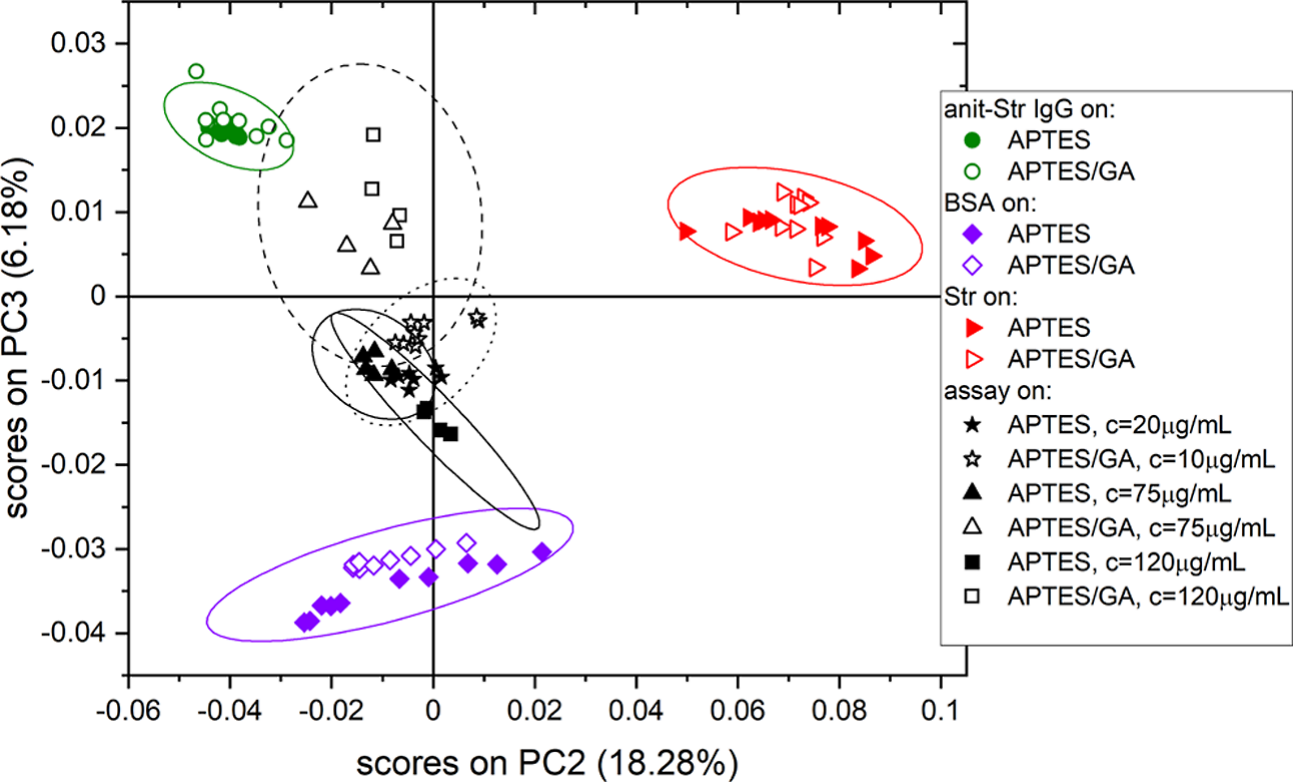
Surface composition analysis with PCA of TOF-SIMS data after completion of the anti-STR IgG/ STR assay protocols on silicon chips modified with APTES and APTES/GA. PC2 vs PC3 score plot for the PCA model involving bare substrates (not presented), the multi-protein (anti-STR IgG, BSA, and STR) layers examined with the WLRS-based biosensor and corresponding to different concentrations of IgG solution during the immobilization step (cf. Figures 2 and S1), and one-component reference layers of anti-STR IgG, BSA, and STR. The ellipses around each of the grouped data points represent the 95% confidence limit. The data points of the one-component layers are centered around three points that can be envisioned as the vertices of a triangle. The composition of any point of the multi-protein layers (mole fraction of IgG, *X*_IgG_, BSA, *X*_BSA_, and STR, *X*_STR_) is expressed by barycentric coordinates (Figure 5), interpreted as 3 masses of pure components placed at the triangle vertices that yield the center of mass at the point location.

**Figure 4:**
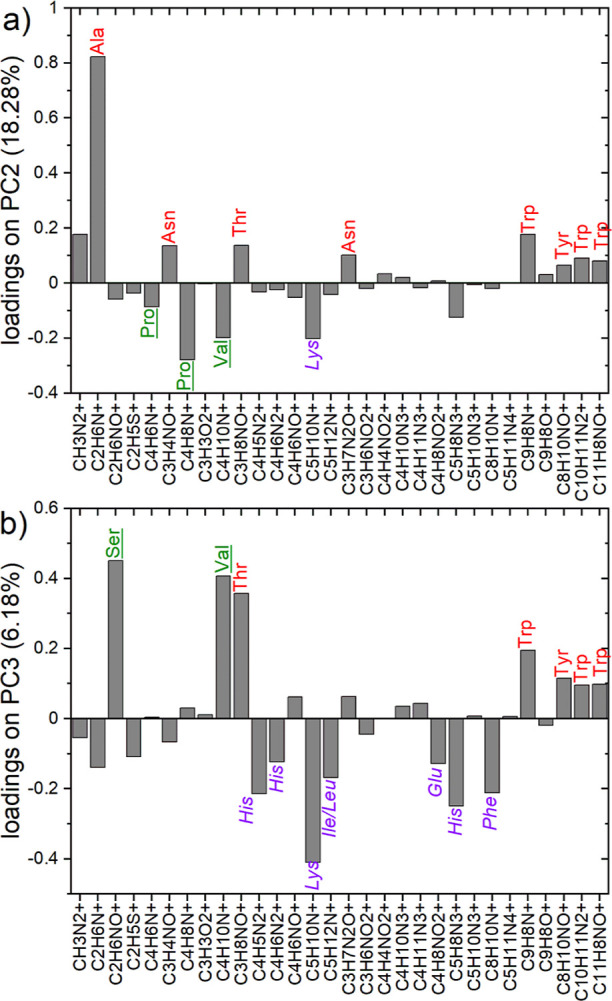
Loading plots for the second (a) and third (b) principal components in the PCA model presented in Figure 3. The ion fragments of amino acids that have higher abundances in IgG, BSA, and STR molecules, respectively, than in other proteins are colored green, violet, and red. For PC2, the ion fragments of amino acids abundant in the STR load in the positive direction, while those abundant in the IgG or BSA load in the negative direction. In turn, PC3 is negatively loaded by ion fragments of amino acids characteristic of BSA and positively loaded by those abundant in IgG or STR.

To enhance chemical specificity of TOF-SIMS, PCA of TOF-SIMS data was performed. The combined TOF-SIMS data, recorded for the multi-protein (anti-STR IgG, BSA, and STR) layers on the APTES and APTES/GA surfaces, the reference layers of component proteins, and bare surfaces, were analyzed. The results of the PCA model, developed for 30 characteristic positive ion fragments of amino acids^34^ (and listed in Figure 4) from over 100 spectra, are presented in Figures 3, 4, and S3. The main source of the variability in the data, expressed by the first principal component PC1 (66.55% of the variance between the samples), can be related to the coverage of the surface with proteins. Indeed, the scores on PC1 (Figure S3b) show that PC1 differentiates bare surfaces from protein layers, and the loadings on PC1 indicate the contributions of the surfaces to some characteristic TOF-SIMS signals of proteins (cf. similar data sets in refs (7) and (8)). In turn, the composition changes described in Figures 3 and 4 by PC2 (capturing 18.28% of variance) and PC3 (6.18%) are independent of those reflected by PC1 due to the orthogonality of principal components. In our subsequent analysis, we assume that the surface density of all (different) proteins on the biochip affects the scores on PC1 but not the scores on PC2 and PC3. Because this issue is central to our analysis, we have performed additional PCA examination (Figure S4a,b) of auxiliary TOF-SIMS data acquired for reference samples corresponding to different surface density of different adsorbed proteins, which confirms this assumption.

The multi-protein surface composition of the biochips is expressed in the score plot (Figure 3) by the principal components PC2 and PC3. The corresponding loading plots (Figure 4) reveal correlations between PC2 and PC3 and the TOF-SIMS intensities of the ion fragments of different amino acids. When the amino acid composition of different proteins is not the same (Table 1),^34,35^ characteristic TOF-SIMS signals can be ascribed to different proteins (Figure 4). Here, the ion fragments of amino acids that have higher abundances in IgG, BSA, and STR, respectively, than in other proteins are marked (Figure 4) in green, violet, and red. Figure 4a shows that PC2 distinguishes between amino acids characteristic of STR, which produce positive loadings, and those abundant in IgG and BSA, which produce negative loadings. In turn, Figure 4b indicates that PC3 differentiates amino acids characteristic of BSA, with negative loadings, from those abundant in IgG and STR, with positive loadings. Therefore, the scores on PC2 and PC3 would describe the abundance in IgG, BSA, and STR of the analyzed multi-protein layer.

**Table 1:** Comparison of (%) Amino Acid Composition of STR,^35^ BSA,^35^ and the Model Antibody^34^

amino acids	STR	BSA	antibody
alanine (Ala)	8.8	8.0	5.1
arginine (arg)	4.9	4.0	2.8
asparagine (Asn)	6.0	2.4	4.6
aspartic acid (asp)	3.5	6.9	4.8
cysteine (cys)	0.0	6.0	2.0
glutamine (gln)	3.1	3.4	4.3
*glutamic acid (Glu)*	4.3	10.1	5.1
glycine (gly)	6.7	2.7	5.4
*histidine (His)*	2.2	2.9	1.9
isoleucine (ile)	2.6	2.4	3.4
*leucine (Leu)*	6.6	10.5	5.9
*lysine (Lys)*	4.0	10.1	6.2
methionine (met)	0	0.7	2.0
phenylalanine (phe)	2.4	4.6	3.9
proline (Pro)	1.0	4.8	6.3
serine (Ser)	5.7	4.8	12.5
threonine (Thr)	16.1	5.7	8.8
tryptophan (Trp)	9.3	0.3	2.0
tyrosine (Tyr)	7.9	3.4	4.6
valine (Val)	5.0	6.2	8.3

**Figure 5:**
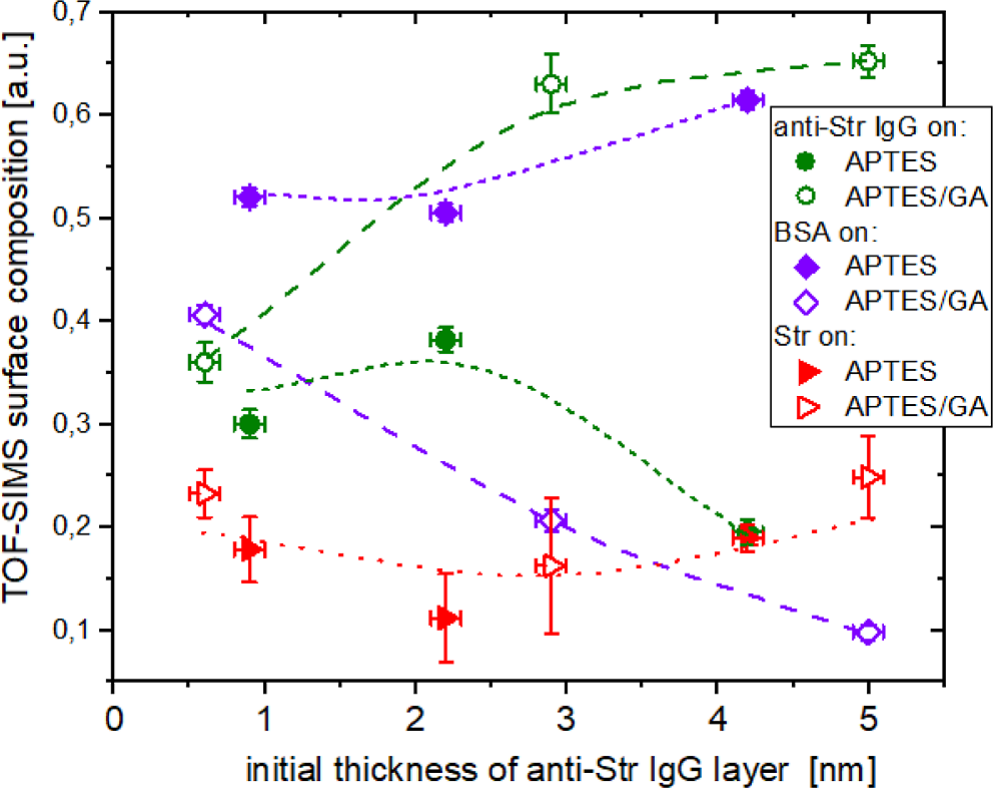
Multi-protein surface composition (anti-STR IgG, BSA, and STR mole fractions) evaluated with PCA of TOF-SIMS data (PC2 vs PC3 scores of Figure 3) for silicon chips modified with APTES and APTES/GA after completion of the anti-STR IgG/STR assay (corresponding to different IgG concentration during the immobilization step) plotted versus the initial WLRS thickness of the IgG layer. Lines are used to guide the eye for protein concentrations of biomolecular layers on APTES/GA (dashed for anti-STR IgG and BSA and dots for STR) and APTES modified chips (short dashed for anti-STR IgG and BSA and dots for STR). Surface composition error bars are propagated standard errors of the mean from several TOF-SIMS measurements of the same (multi-component or pure component) sample.

Consequently, the data points in the PC2 vs PC3 score plot (Figure 3), corresponding to reference layers of the proteins involved (anti-STR IgG, BSA, and STR) are well separated (this feature is mimicked by the additional PCA analysis of different proteins adsorbed with different surface densities, Figure S4b). The data points of the pure component layers are centered around three points that can be envisioned as the vertices of a triangle. Because principal components (PCs), for TOF-SIMS data, exhibit linear correlation with organic surface composition^36,37^ (and the latter is expressed as a molar concentration for molecular mixtures^38^), the assumption that the scores on PC2 and PC3 vary linearly with mole fraction of IgG, BSA, and STR (*X*_IgG_ + *X*_BSA_ + *X*_STR_ = 1) is made. Accordingly, the composition of any point on the PC2 vs PC3 score plot (Figure 3) can be expressed by barycentric coordinates, interpreted as 3 masses (*X*_IgG_, *X*_BSA_, and *X*_STR_) of pure components placed at the triangle vertices that yield the center of mass at the point location. For this purpose, the triangle vertices are defined by average values of scores on PC2 and PC3 for pure component reference layers regardless of the substrate modification (APTES or APTES/GA). The barycentric coordinates are then calculated for each multi-protein layer on biochips based on the average values of scores on PC2 and PC3 for different point groups of the PC2 vs PC3 score plot (Figure 3). The obtained barycentric coordinates are plotted in Figure 5 as surface composition (IgG, BSA, and STR mole fractions) for the multi-protein layers of the anti-STR IgG/STR assays executed on APTES or APTES/GA surfaces for three different surface densities of immobilized anti-STR IgG molecules. All multi-protein layers have roughly similar STR concentration, but those on APTES/GA are richer in IgG and less in BSA than those on APTES, with the disparity between IgG and BSA concentrations growing as the initial thickness *d*_Γ_ of the IgG layer increases (cf. Figures 3 and 5). Because the WLRS-based biosensor response reflects the cumulative mass loading of all proteins, the surface composition of multi-protein layers is converted from mole to mass fractions (with *M*_w_ of 53, 66, and 150 kDa taken for STR, BSA, and IgG, respectively). Then, the mass fractions are multiplied by the total biomolecular layer thickness *d*_Γ_ determined with WLRS after completion of any of the anti-STR IgG/STR assays (Figures 2a,b and S1) to provide the TOF-SIMS estimations of the layer thickness for the IgG (Section 3.3) and STR components (Section 3.4).

### 3.2 IgG Molecule Immobilization: In-Flow Approach on APTES and APTES/GA Surfaces

**Figure 6:**
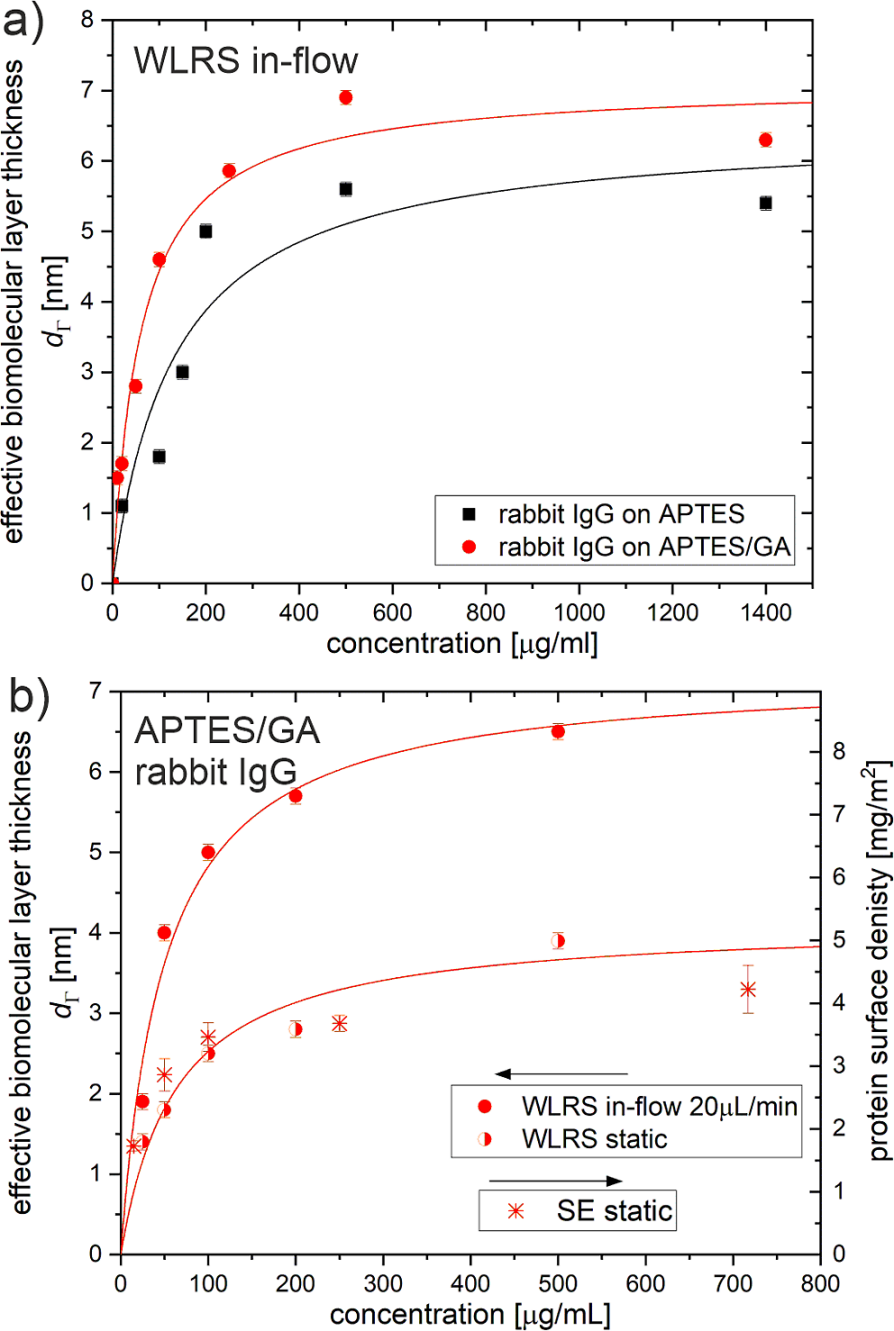
(a) Adsorption isotherms for in-flow immobilization of rabbit IgG on silicon chips modified with APTES (black squares) and glutaraldehyde activated APTES (APTES/GA) (red circles). The effective thickness of the biomolecular layer *d*_Γ_ was determined with the WLRS-based biosensor after the immobilization step was completed. (b) Adsorption isotherms of rabbit IgG on silicon chips modified with APTES/GA under in-flow (solid circles) or static conditions (half-filled circles, asterisks). The surface amount of immobilized IgG molecules is represented by the effective thickness *d*_Γ_ of the biomolecular layer determined with WLRS for the chip area located under the microfluidic cell of the WLRS-based biosensor (solid and half-filled circles, left axis) or by the protein surface density determined with SE for the chip washed with water and dried after solution droplet deposition (asterisks, right axis). The lines in (a,b) describe the experimental data on the basis of the Langmuir model.

After different molecules involved in the model (capture) assay have been resolved with TOF-SIMS, we can analyze the situation after each step of the examined assay procedures (immobilization of IgG molecules, blocking step, and immunoreaction). For the first step, we compared the physical adsorption and covalent coupling of proteins on APTES-modified surfaces under flow conditions. For this purpose, the solution of IgG molecules (anti-STR IgG or rabbit IgG) was run over the modified Si/SiO_2_ chips with a constant flow of 20 μL/min for 20 min. The adsorption isotherms presented in Figure 6a for rabbit IgG and in Figure S5 for anti-STR IgG show the saturation value of the effective biomolecular layer thickness *d*_Γ_ at the end of the flow of the IgG solution plotted as a function of the solution concentration. The fitting of the Langmuir model to the adsorption isotherms for rabbit IgG provided the values of surface binding capacity of about ∼6.5 nm and ∼7.1 nm and affinity constant of about ∼1.1 × 10^6^ 1/M and ∼2.5 × 10^6^ 1/M for immobilization on APTES and APTES/GA, respectively. Additionally, a comparative examination of static and in-flow immobilization was performed using the fluidic module of the WLRS platform on APTES/GA modified silicon chips. For this purpose, several solutions of rabbit IgG with concentrations in the range of 10–500 μg/mL were used for both static and in-flow immobilization experiments. In-flow immobilization was performed by running the IgG solution over the chip for 15 min, while for static approach the syringe pump was turned off immediately after filling of the fluidic cell with the IgG solutions, and the chip was incubated for 15 min. After completion of the incubation with the IgG solutions all chips were washed by flowing PBS buffer. The effective thickness of the IgG layers determined with WLRS directly after performing the immobilization is compared in Figure 6b for static and in-flow approaches. As shown, the in-flow immobilization results, over the entire concentration range, in a more thick protein layer than that obtained from the static approach. This difference is more pronounced for high concentrations of solution (more than 200 μg/mL). The fitting of the Langmuir adsorption isotherm to static immobilization data gives a binding capacity of about ∼4.0 nm, which is significantly lower than the value obtained under in-flow conditions, and an affinity constant of about ∼2.3 × 10^6^ 1/M. In addition, the rabbit IgG surface density values (in mg/m^2^) determined with spectroscopic ellipsometry (SE) for immobilization of IgG by droplet deposition of the solution were juxtaposed in Figure 6b with the effective layer thickness *d*_Γ_ of WLRS (in nm) under static and in-flow immobilization conditions. The scaling that provides the best coverage of points corresponding to both static immobilization experiments provides *a* factor of about 1.28 between the effective layer thickness *d*_Γ_ determined from the WLRS measurements to the surface density Γ obtained from SE measurements. Therefore, this value, corresponding also to protein density (1.28 g/cm^3^),^7,39^ was taken into account to estimate protein surface density from WLRS data.

### 3.3 Surface Binding Capacity under In-Flow Conditions

**Figure 7:**
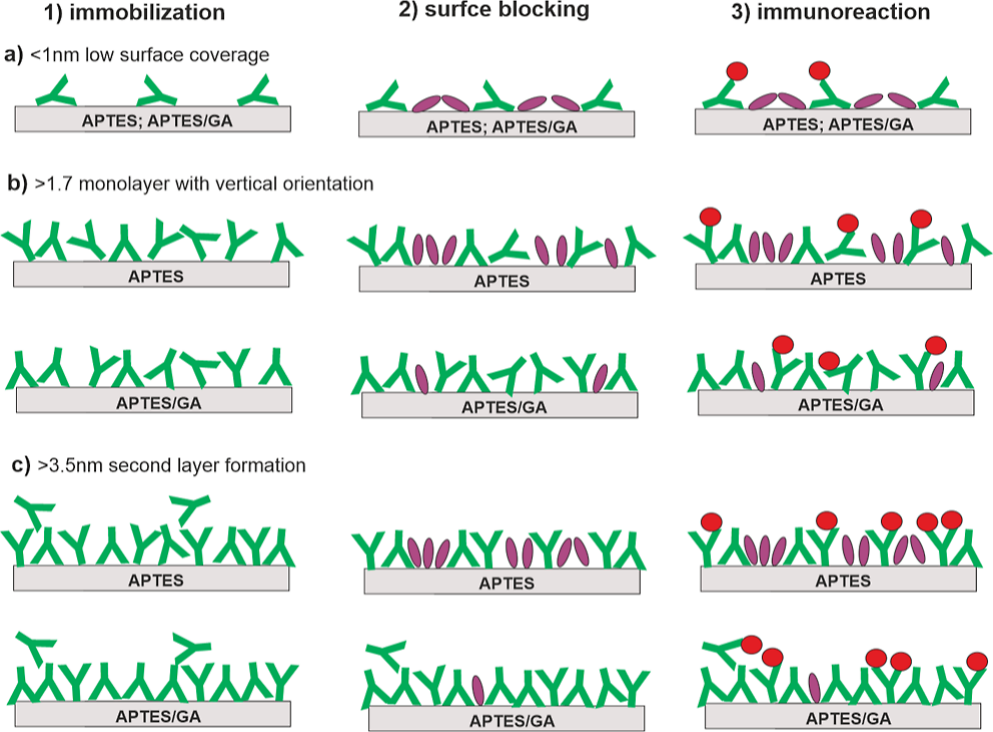
Scheme of the arrangement of the molecules after the subsequent steps (1–3) of the anti-STR IgG/STR assays carried out on APTES and APTES/GA surfaces, starting with different concentrations of IgG solution and therefore different effective initial thicknesses *d*_Γ_ of the IgG layer after the immobilization step (a–c). (a) For low surface coverage with IgG antibodies, the protein monolayer is completed with BSA during the blocking step, for both APTES and APTES/GA modified surfaces. The high molar binding ratio of STR to anti-STR IgG (mbr ∼0.7) reflects this side-on orientation. (b) When the monolayer of IgG molecules is completed, the antibodies physically adsorbed on APTES rather than those covalently bound on APTES/GA are partially replaced with BSA during blocking. A lower STR/ anti-STR IgG molar binding ratio (mbr ∼0.4) is determined corresponding to dominant vertical orientation and partial exchange of IgG molecules with BSA. For physical adsorption on APTES, the lower amount of IgG molecules because of immobilization instability is compensated by a higher fraction of the exposed Fab domain, which results in comparable binding stoichiometry for both surface modifications. (c) A second layer of IgG molecules is formed during immobilization on APTES and APTES/GA surfaces which is completely or substantially reduced during blocking. As for the complete underlying monolayer (b), the physically adsorbed antibodies are partially exchanged with BSA leading to a reduction of the observed binding stoichiometry.

To properly interpret the WLRS data, its original observable *d*_Γ_ should be considered only as the effective thickness of the biomolecular layer, reflecting the cumulative surface density Γ of *all* different proteins on the biochip. An in-depth analysis of the vertical arrangement of molecules immobilized on APTES and APTES/GA was provided earlier^7,8^ by TOF-SIMS, which combines a higher sensitivity for the outermost nanometer regions of probed proteins combined with detectable differences in amino acid composition of the different protein domains. In particular, information on the inner structure of biomolecular layers was provided by the determined relationship between antibody orientation and its surface density Γ,^7,8^ which is properly described by random rather than close-packed molecular arrangement. The situation is consistent with a lower molecular packing efficiency (jamming limit, Θ_∝_ ∼ 0.55) due to random sequential adsorption.^6^ The Langmuir model can provide a useful reference to the observed adsorption isotherms (Figure 6) with a binding capacity equal to (Γ_ind_Θ_∝_), reflecting the (maximum) mass loading Γ_ind_ of the individual IgG molecule.^7,8^ The inner structure of immobilized IgG molecules (Figure 7) depends on surface density and immobilization scheme. A decrease in the surface area accessible to each molecule forces a more vertical IgG orientation with the Γ_ind_ values critical for each specific orientation provided by geometric considerations.^5,40^ The transition from flat-on to side-on and up to vertical alignment is expected every time the surface density Γ reaches a critical (Γ_ind_Θ_∝_) value (rescaled to *d*_Γ_ ∼1 and 1.7 nm^7,8^) and consistent with other reports, e.g., ref (18) and (19). For vertical alignment, the immobilization scheme determines the proportions of IgG molecules that adapt the coexisting orientations of tail-on and head-on.^7,8^

The adsorption isotherms for in-flow immobilization of IgG antibodies (Figures 6a and S5) indicate that the surface binding capacity obtained by covalent coupling on APTES/GA is approximately 15% larger than that achieved employing physical adsorption to APTES. This is consistent with previous studies on antibody immobilization under static conditions, performed by droplet deposition of the protein solution, which reported a slightly higher surface amount of IgG molecules immobilized on glutaraldehyde activated APTES surfaces compared to those modified only with APTES.^17,18^ It is worth noting that applied glutaraldehyde aqueous solution under neutral pH conditions contains various glutaraldehyde forms such as monomeric dialdehyde, cyclic hemiacetal, and different reactive polymeric forms.^41,42^ The presence of the several molecular forms of glutaraldehyde leads to different possible mechanisms of the reaction with proteins that increases its reactivity toward proteins as compared to the monomeric glutaraldehyde.^41^

In turn, the comparison made for the APTES/GA surface (Figure 6b) shows that in-flow immobilization results in about 1.7 times higher surface binding capacity (7.1 nm) than static adsorption (4 nm). A similar conclusion emerges when the binding capacity, determined (6.5 nm) for the APTES surface, is compared to the reported WLRS thickness *d*_Γ_ (∼3 nm) of immobilized antibodies.^43^ The formation of more developed protein adlayers under flow conditions can be related to a continuous supply of protein molecules, while a depletion of the surface solution concentration is expected for static adsorption.^9^ Moreover, an increase in protein saturation coverage with increasing flow rate was reported and was related to hindered interfacial relaxations of molecules.^44^

Finally, the juxtaposed WLRS and SE data (Figure 6b) reveal the scaling factor (1.28 g/cm^3^) to estimate, from the WLRS thickness *d*_Γ_, the protein surface density Γ and therefore the expected structure of the immobilized IgG molecules,^5,7^ as outlined on Figure 7. The adsorption isotherms (Figure 6a) indicate that in-flow immobilization can provide the surfaces with the amount of IgG adjusted from low coverage (*d*_Γ_ < 1 nm) to monolayers (with vertically oriented antibodies for *d*_Γ_ > 1.7 nm) and to bilayers (*d*_Γ_ > 3.5 nm).

### 3.4 Surface Blocking: Evaluation of the Partial Desorption of IgG Molecules

**Figure 8:**
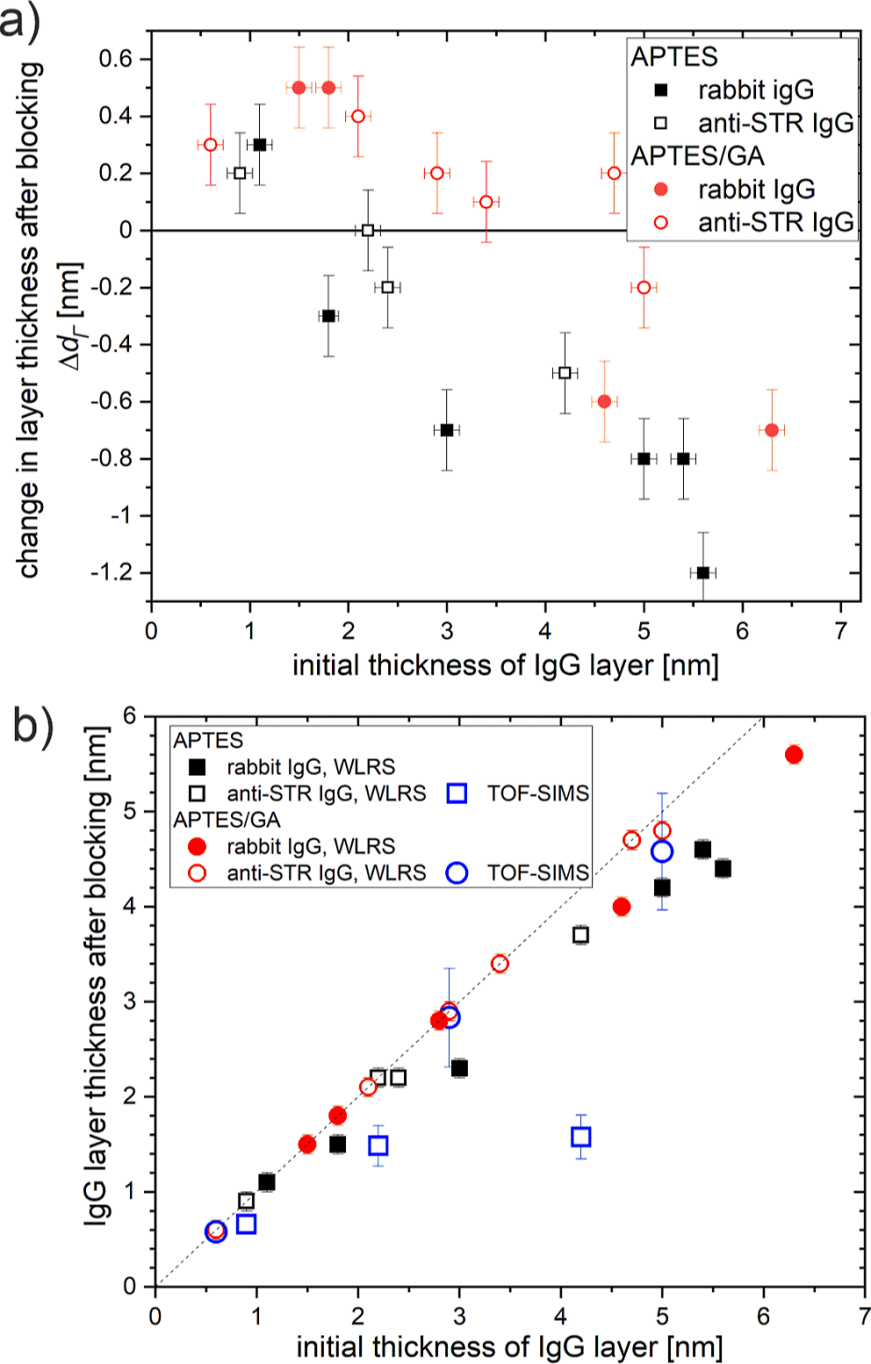
The effect of the blocking step on the effective thickness of biomolecular layers for silicon chips modified with APTES or APTES/GA with immobilized rabbit IgG or goat anti-STR IgG molecules. Blocking involved washing with PBS buffer, running of BSA solution, and again washing with PBS buffer after the in-flow immobilization of IgG molecules was completed. (a) Change Δ*d*_Γ_ in the effective thickness of the biomolecular layer, as determined with the WLRS-based biosensor before and after the blocking procedure, plotted versus the initial layer thickness of rabbit IgG (solid symbols) and goat anti-STR IgG molecules (open symbols) on silicon chips modified with APTES (black squares) or APTES/GA (red circles), respectively. (b) Effective thickness of the IgG layer after the blocking step plotted versus the initial thickness of rabbit IgG (solid symbols) and goat anti-STR IgG layer (open symbols) on the silicon chips modified with APTES (squares) or APTES/GA (circles). The black and red symbols denote the results deduced from the WLRS data under the assumption that the negative changes in Figure (a) represent the amount of desorbed IgG molecules. In turn, the blue symbols denote the TOF-SIMS estimations of the IgG layer thickness, obtained as the product of the IgG mass fraction (based on the mole fraction from the PCA analysis of TOF-SIMS data) and the total thickness of the biomolecular layer (from WLRS), determined after completion of the anti-STR IgG/ STR protocol.

Second, blocking of free surface sites is a necessary step in the biofunctionalization of a sensor surface. For this purpose, an inert protein such as BSA is commonly applied. In the course of the examined immunoassay protocols, the chips, after completion of the immobilization of the IgG molecules, were washed by running PBS buffer and subsequently blocked by running a BSA solution (2 mg/mL in PBS) for 15 min and again washed with PBS, what we refer to in this paper as the blocking step. The change in the effective thickness of the biomolecular layer on chips upon the blocking step was monitored with WLRS for both assay configurations (anti-STR/STR and IgG/anti-IgG) and different concentrations of the IgG solution used for immobilization. This allows for evaluation of the blocking step impact with respect to the initial surface amount of immobilized IgG molecules. Representative real-time responses of the WLRS sensor are presented in Figures 2 and S1. For each assay protocol, the change Δ*d*_Γ_ in the effective thickness of the adlayer during the blocking step was determined from the WLRS data. These data are plotted in Figure 8a as a function of the initial thickness of IgG molecules layer, as we determined immediately after the completion of the immobilization step. Furthermore, based on the assumption that the negative Δ*d*_Γ_ changes in *d*_Γ_ represent the amount of desorbed IgG, we calculated the effective thickness of the IgG layer after the blocking step which is shown in Figure 8b. Alternatively, positive Δ*d*_Γ_ changes correspond to adsorption of BSA molecules onto the chip surface.

### 3.5 Immobilization Stability on APTES and APTES/GA Surfaces

The results presented above show that the immobilization stability of IgG antibodies is affected during the blocking procedure in a way that depends on the immobilization scheme, i.e., if physical adsorption or glutaraldehyde covalent coupling has been used (Figure 8). The first effect that influences the immobilization stability is molecular desorption, demonstrated as a reduction in the thickness *d*_Γ_ of the biomolecular layer observed with WLRS during BSA blocking. For the APTES/GA surfaces, a slight increase in *d*_Γ_ is observed for layers with small initial *d*_Γ_ values (Figure 8a), whereas a reduction is observed for thicker initial IgG layers (*d*_Γ_ > 3.5 nm). This is interpreted as a change from adsorption of BSA molecules to partial desorption of IgG molecules when IgG bilayers are formed during immobilization (Figure 7). This explanation is supported by the agreement between the WLRS signals (red circles in Figure 8b) and the TOF-SIMS estimations (blue circles in Figure 8b) of the IgG layer thickness after blocking, obtained for a wide range of *d*_Γ_ values (0 < *d*_Γ_ < 5.5 nm). Reversible protein binding to the second layer has previously been reported and is related to a weak protein–protein interaction as compared to strong covalent coupling to surface functional groups.^5,9^

In contrast, for APTES surfaces, physically adsorbed IgG molecules desorb upon the flow of the buffer and BSA solution starting from initial IgG thickness values *d*_Γ_ > 1.7 nm (Figure 8a). This value can be recognized as the characteristic value corresponding to monolayer formation with vertically aligned IgG molecules.^7,8^ The different *d*_Γ_ values of the onset of molecular desorption for APTES/GA and APTES reflect different effective molecule–surface interactions that are also dependent on the orientation of immobilized molecules (Figure 7). Also, the flow of solution over the protein layer enhances the desorption of physically adsorbed protein molecules. For proteins adsorbed to APTES under static conditions, blocking-induced desorption was observed when the bilayers were formed.^15^

**Figure 9:**
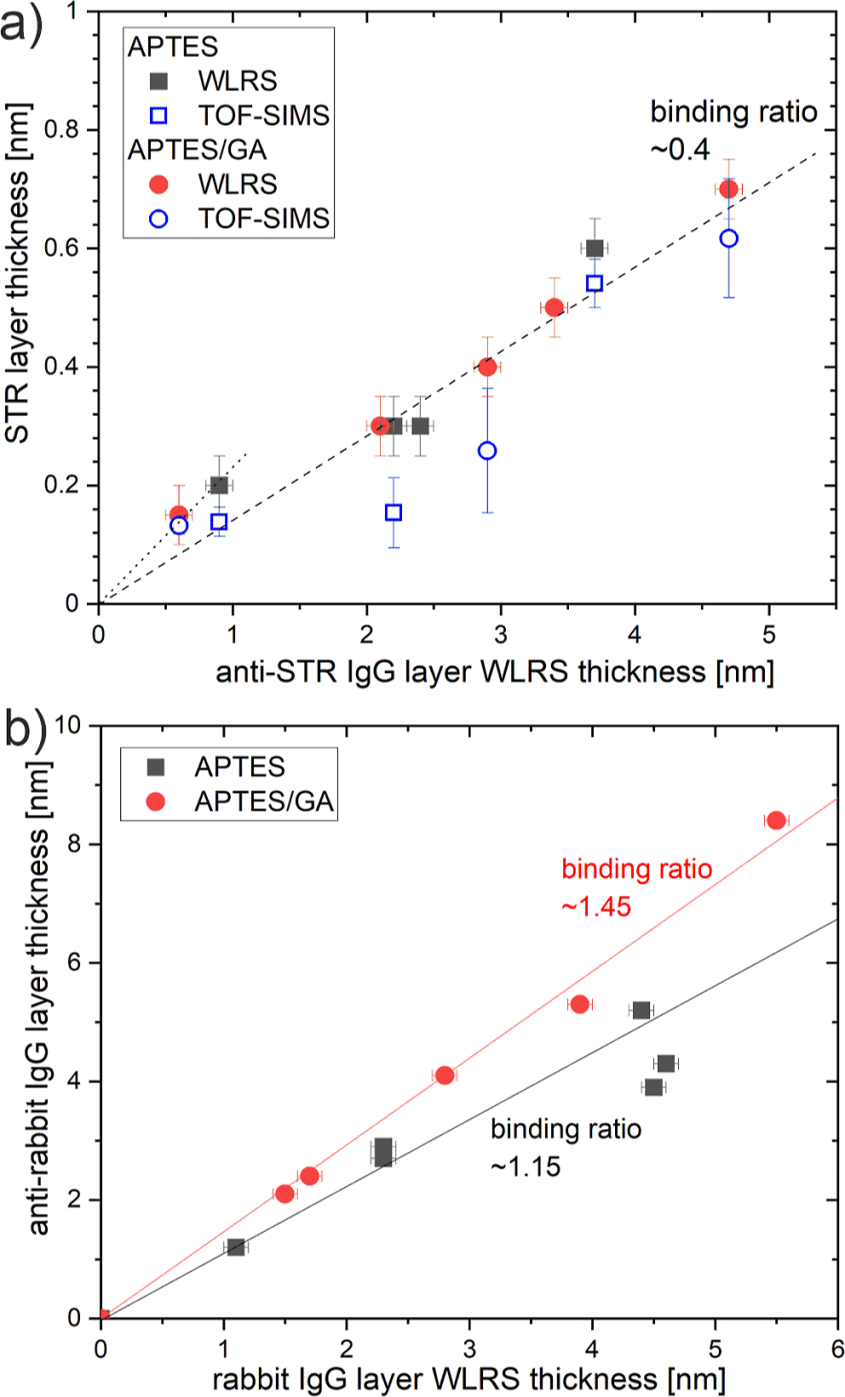
Analysis of immunoreactions on the APTES (squares) and APTES/GA surfaces (circles): the effective layer thickness of the STR antigen bound during the anti-STR IgG/STR capture assay (a) and the anti-rabbit IgG antibody bound during the IgG/anti-IgG direct binding assay (b), plotted versus the effective thickness *d*_Γ_ of the IgG layer, determined by WLRS after the completion of the blocking step (data abscissae correspond to data ordinates of Figure 8b). An increase in the WLRS signal after immunoreaction is taken as a measure of the layer thickness of STR (a) and anti-rabbit IgG (b), marked with black and red symbols. In turn, the blue symbols denote the TOF-SIMS estimations of the STR layer thickness (a), calculated as the product of the STR mass fraction (based on the mole fraction from the PCA analysis of TOF-SIMS data) and the total thickness of the biomolecular layer (from WLRS), determined after completion of the anti-STR IgG/STR protocol. The slopes reflect the WLRS estimations of the binding stoichiometry in the immune complexes formed for different IgG surface amounts (a) and different immobilization schemes (b), expressed as the molar binding ratio of STR to anti-STR IgG (a) and anti-IgG to IgG (b).

In turn, the data acquired for the APTES surfaces (Figure 9b) indicate a second effect that affects the immobilization stability of IgG antibodies, that is, the partial exchange of IgG antibodies with BSA molecules for adlayers with *d*_Γ_ > 1.7 nm. This effect is reflected by the disparity between the TOF-SIMS estimations (blue squares in Figure 8b) of the IgG layer thickness *d*_Γ_ after blocking and the much higher WLRS thickness values (black squares in Figure 8b), which account only for molecular desorption. Although TOF-SIMS data indicate partial exchange of antibodies with BSA molecules for APTES (not reflected in negative Δ*d*_Γ_), such an effect is negligible for APTES/GA (when Δ*d*_Γ_ = 0).

### 3.6 Capture and Direct Assays: Binding Stoichiometry Evaluated with WLRS

Third, we examined the efficiency of the immunoreaction based on the WLRS responses during the model capture and direct binding assays, that correspond to an increase in the effective thickness *d*_Γ_ of the biomolecular layer, as presented in Figures 2 and S1. For the anti-STR/STR (model capture) assay, the immunoreaction step was performed by flowing 10 μg/mL STR solution for 15 min. For the IgG/anti-IgG (model direct binding) assay, a 10 μg/mL solution of goat anti-rabbit IgG was passed over a biofunctionalized chip for 20 min. On the basis of the changes in *d*_Γ_, registered after the same time of the solution flow, the apparent layer thicknesses of STR and anti-IgG were determined. Such experiments were performed for both types of silicon chip modification and for various concentrations of the IgG solution, used in the immobilization step. Additional experiments, shown in Figure S2 in Supporting Information, excluded non-specific adsorption to the biomolecular layers after blocking step. The thickness values corresponding to the specifically bound onto chip molecules are presented in Figure 9a,b for the anti-STR/STR and IgG/anti-IgG assay configurations, respectively, as a function of the effective WLRS thickness of the IgG layer prior to immunoassay (data abscissae correspond to the data ordinates of Figure 8b). Furthermore, the apparent binding stoichiometry of the immune complexes formed onto the chips for both assay configurations was evaluated. The WLRS estimations of this quantity are marked as the slopes in Figure 9a,b, respectively, reflecting the average molar binding ratio of STR to anti-STR IgG and anti-IgG to IgG. For the single immunoreaction the molar binding ratio was calculated as the ratio of the WLRS thickness of the specifically bound molecules (STR or anti-rabbit IgG, respectively) to the corresponding WLRS thickness of the surface immobilized IgG molecules (anti-STR IgG or rabbit IgG, respectively), each divided by the molecular weight of that particular molecule (53 kDa for STR and 150 kDa for all IgG molecules). Different estimations of binding stoichiometry are determined for various IgG surface amounts, when the anti-STR/STR assay was applied (Figure 9a), and for various immobilization schemes, when the IgG/anti-IgG assay was used (Figure 9b). For the model capture assay, a STR/anti-STR IgG binding ratio of about 0.4 was calculated for IgG layer WLRS thickness values *d*_Γ_ > 1.7 nm, and a higher ratio (about 0.7) for lower values of *d*_Γ_. Also, no differences are indicated in the efficiency of STR antigen binding when the antibodies have been immobilized by physical adsorption or covalent coupling. In turn, singular values for the entire *d*_Γ_-range of the binding stoichiometry are determined for the model direct assay. The anti-IgG/IgG binding ratio values are equal to 1.15 and 1.45, respectively, for the physically adsorbed and covalently bound rabbit IgG molecules.

### 3.7 Direct Binding Assay: Effect of Immobilization Stability on APTES and APTES/GA Surfaces

The response of the WLRS biosensor to the direct binding assay (Figure 9b) reveals a single value of the molar binding ratio of anti-IgG to IgG for the entire range of initial *d*_Γ_ values. These values of the binding ratio depend on the immobilization approach and are equal to 1.45 and 1.15, respectively, for the APTES/GA and APTES modified surfaces. Similar observations have been made for a direct binding assay with IgG molecules physically adsorbed on dimethylsilane—modified silicon surfaces and covalently coupled on APTES/GA.^45^ In the IgG/anti-IgG (model binding direct) assay, surface-immobilized rabbit IgG molecules play the role of antigens with the epitopes for polyclonal anti-rabbit IgG antibody binding located on both the Fc and F(ab)_2_ domains. Therefore, in contrast to the anti-STR/STR (model capture) assay, the orientation of surface-immobilized IgG molecules does not affect the binding stoichiometry of the immune complexes formed during the IgG/anti-IgG (direct binding) assay. Because the orientation of IgG depends crucially on the immobilization method and the thickness *d*_Γ_ of the protein adlayer (surface density),^7,8^ other mechanisms that involve these two factors should be considered. The impact of the surface density of IgG molecules, through increasing steric hindrance, on the binding stoichiometry is not decisive in light of its single values for the entire *d* range (Figure 9b). In turn, the different WLRS values of the anti-IgG/IgG binding ratio for APTES/GA (1.45) and APTES (1.15) point to different immobilization stability between IgG molecules adsorbed physically and coupled covalently to the surface.

The molecular desorption prior to immunoreaction, discussed in Section 3.4, is monitored with WLRS (Figure 2cd, before step 3) and is reflected in the *d*_Γ_ values used to evaluate the binding ratios (Figure 9). On the contrary, the molecular exchange of BSA and IgG, the second effect that affects the immobilization, is not resolved by WLRS and is not accounted for in the binding stoichiometry. The binding stoichiometry is determined from the WLRS response of bound anti-IgG and surface immobilized IgG (Figure S6). Because the molecular exchange is negligible for APTES/GA, we take the corresponding value (1.45) as the real ratio of the bound anti-IgG to the surface immobilized IgG. In turn, for APTES the WLRS response of the bound anti-IgG is taken with respect to that of the surface-immobilized IgG, which reflects not only the IgG molecules but also the BSA molecules exchanged with IgG during the blocking step (Figure S6). Therefore, the ratio of both WLRS responses is lower (1.15). Thus, using both WLRS values of the anti-IgG/IgG binding ratio (see Section S5), it is calculated that about 12% of physisorbed IgG molecules are exchanged by BSA molecules.

### 3.8 Capture Assay: Effect of Dominant Antibody Orientation Included

In turn, the responses of the WLRS biosensor to the anti-STR/STR (model capture) assay (solid symbols in Figure 9a) reveal the same values of binding stoichiometry for both the non-covalent (APTES) and the covalent (APTES/GA) immobilization scheme. These WLRS values of the STR/anti-STR molar binding ratio, marked as the distinct slopes in Figure 9a, are equal to 0.4 for IgG layers with *d*_Γ_ > 1.7 nm and about 0.7 for lower values of *d*_Γ_. For low surface coverage with IgG antibodies, a side-on orientation of IgG is expected^7,8^ for both APTES and APTES/GA surfaces, and the determined WLRS molar binding ratio is comparable to the value of 0.4–0.7 reported for the IgG/anti-IgG ratio^7^ (in accordance with the theoretical maximum value of 1). However, for IgG layers with *d*_Γ_ > 1.7 nm, monolayers of vertically oriented antibodies have been reported^7,8^ with inner structure dependent on the immobilization method. In particular, the proportions of molecules that adapt tail-on and head-on alignment are 1:3 for APTES/GA and 1:1 for APTES.^8^ The WLRS value of the STR/anti-STR molar binding ratio of 0.4 is apparently identical for both immobilization methods (Figure 9a), but in fact it reflects the actual ratio only for the APTES/GA modified surfaces. In turn, partial replacement with BSA of the vertically aligned antibodies bound to APTES, with a fraction of exposed Fab domains higher than that of APTES/GA, must be assumed to explain the finding that the observed binding stoichiometry is the same. Also, by applying a 12% level of exchange of immobilized IgG molecules with BSA molecules a value of 0.6 is obtained for the STR/anti-STR molar binding ratio onto the APTES surface. Hence, both actual ratios, 0.4 for APTES/GA and 0.6 for APTES, correspond to the theoretical maximal values of 0.5 for 1:3 and 1 for 1:1 proportion of molecules with tail-on and head-on alignment. In fact, the observed binding stoichiometry is usually lower than the predicted maximal binding ratio, because of steric hindrance between captured antigens, which strongly depends on the antigen size. For example, our recent studies reported an IgG/anti-IgG ratio of approximately 0.2 for the 1:3 and 0.4 for the 1:1 proportion.^7^ These binding stoichiometry values are smaller than those reported here, since they have been obtained for an antigen (IgG) larger than STR (*M*_w_ ∼ 150 kDa for IgG vs *M*_w_ ∼ 53 kDa for STR).

The PCA analysis of the TOF-SIMS data acquired *after* the completion of the anti-STR/STR (model capture) assay reveals that all multi-protein layers have a similar mole fraction of STR, and these on APTES/GA are more rich in IgG and less rich in BSA than those on APTES, with the disparity between IgG and BSA composition growing with *d*_Γ_ (Figure 5). These results provide, together with the total layer thickness *d*_Γ_, the TOF-SIMS estimations (Section 3.1) of the effective layer thickness of STR (open symbols in Figure 9a), which for both APTES and APTES/GA surfaces are hardly different from those calculated based on the real-time WLRS responses (solid symbols in Figure 9a) due to the reaction of the immobilized antibodies with the STR antigen (after step 3, Figures 2a,b and S1). Also, the respective TOF-SIMS estimations of the effective IgG layer thickness (blue symbols in Figure 8b) are juxtaposed with the WLRS responses (red and black symbols in Figure 8b) obtained *directly* after blocking (before step 3, Figures 2a,b and S1). Although the WLRS and TOF-SIMS data match each other for APTES/GA, they disagree for APTES modified surfaces. The comparisons presented above of the data obtained directly (WLRS) and indirectly (TOF-SIMS), before (WLRS) and after (WLRS, TOF-SIMS) the capture assay, confirm that the molecular exchange of the immobilized IgG antibodies occurs during blocking (for non-covalent immobilization) and not during the immunoreaction (STR capture). Previously, partial molecular exchange of antibodies with blocking molecules, BSA^14,16^ or milk proteins^17^ or with the albumin antigen conjugate,^14,16^ physically adsorbed to the APTES surface, has been observed during immunoreaction.^14,16^ These effects were revealed with TOF-SIMS and not by biosensor response, leading to an inaccurate evaluation of the binding stoichiometry when based only on the real-time responses of integrated Mach–Zehnder interferometric biosensors onto silicon chips.^14^ The above analysis points out that, due to molecular replacement occurring for biomolecules immobilized by physical adsorption, the binding stoichiometry cannot be accurately determined from the response of the biosensor since it corresponds to the cumulative mass loading of surface-immobilized and assay-bound biomolecules.

## 4 Conclusions

In this work, we analyzed the *in-flow biofunctionalization and assay* on aminosilanized silicon chips implementing the microfluidic module of the WLRS—based optical biosensor. In addition to the adlayer thickness *d*_Γ_, monitored in real time with WLRS, the *multi-protein composition* was examined by TOF-SIMS after the completion of the assay. This is the first attempt to resolve the different components of biofunctionalization and assay, which were all introduced on the sensor chip by the flow of various solutions, that is, they were all deposited in situ using the most explored *in-flow* strategy.^26,28^ Two strategies of IgG antibody immobilization, *physical adsorption* (APTES) and glutaraldehyde *covalent coupling* (APTES/GA), followed by blocking of free surface sites with BSA, were compared through a model STR *capture assay*. Also, a *direct binding* anti-IgG *assay* was examined to contrast between surface-bound IgG molecules acting as antibodies or antigens. The IgG adsorption isotherms, determined with WLRS and juxtaposed with SE data, reveal that *in-flow immobilization* is ∼1.7 times more efficient than its static counterpart, resulting in surfaces with the amount of IgG adjusted from low coverage to monolayer completion and up to second layer formation.

The *multi-protein* surface *composition* (IgG, BSA, and STR) was evaluated by TOF-SIMS combined with PCA (Section 3.1). To this end, a novel approach was introduced by applying barycentric coordinates to the PCA score plot, where the reference points of pure proteins formed the triangle vertices. The estimations provided by this approach for layer thickness of the IgG component agree with WLRS data for APTES/GA, confirming the assumption on linear correlation between PCs and composition.^37^ This approach extends to a more semi-quantitative level the PCA analysis of three-component multi-molecular surfaces analyzed with TOF-SIMS.^46^

The results of TOF-SIMS complement the real-time data obtained with the WLRS biosensor and resolve different factors that affect *immobilization stability* and *binding stoichiometry*, reflected in the biosensor response upon blocking and assay, respectively. While previous TOF-SIMS studies have examined only the effects affecting the biosensor response to an assay,^14,17^ the scope of this work also covers the response upon blocking and further examines how the factors affecting each of both responses are interrelated. The combination of WLRS and TOF-SIMS shows that *immobilization stability* is affected by molecular desorption and molecular exchange to different extent depending on the immobilization strategy and the amount of surface bound antibodies (Sections 3.4 and 3.5), the latter limited by surface binding capacity (Section 3.3). The WLRS biosensor responses augmented with the respective TOF-SIMS data indicate that the stability of immobilization is affected during the blocking and not the assay, and resolves its two components, molecular desorption and exchange between different molecules (Sections 3.4 and 3.5). Depending on the effective interactions between the molecule and the surface, antibody desorption occurs when monolayers of vertically oriented molecules (APTES) or molecular bilayers (APTES/GA) are formed. In turn, the *binding stoichiometry* revealed by WLRS for the capture assay is influenced by the *stability of immobilization* and the orientation of surface-bound antibodies (Section 3.6), which has previously been determined by TOF-SIMS^7,8^ as a function of the surface amount of IgG molecules for both immobilization methods. Furthermore, the WLRS analysis of a direct binding assay decouples the impact of immobilization stability on binding stoichiometry determined from that of orientation of surface-bound IgG molecules (Section 3.8).

## Supporting Information Available

WLRS sensor responses for anti-STR IgG/STR capture assay; examination of the nonspecific adsorption during assay; PCA model applied to TOF-SIMS data to examine the multi-protein surface composition ; PCA model applied to TOF-SIMS data to examine one-component protein layers with different surface densities; adsorption isotherms for in-flow immobilization of anti-STR goat IgG; and degree of IgG molecules exchanged with BSA determined from the direct assay monitored with WLRS (PDF)
