# Supplementary material for: Covalent and Non-covalent In-Flow Biofunctionalization for Capture Assays on Silicon Chips: White Light Reflectance Spectroscopy Immunosensor Combined with TOF-SIMS Resolves Immobilization Stability and Binding Stoichiometry
Source: Langmuir. 2023 Jul 12;39(29):10216–29. doi: 10.1021/acs.langmuir.3c01181 (PMC10373486; doi:10.1021/acs.langmuir.3c01181)
Supplement: Supplementary file 1 — la3c01181_si_001.pdf [file la3c01181_si_001.pdf]

Supplementary Material

Covalent and non-covalent in-flow biofunctionalization for capture assay on silicon chips: White Light Reflectance Spectroscopy immunosensor combined with TOF-SIMS resolves immobilization stability and binding stoichiometry

Katarzyna Gajos[a*], Alicja Orzech[a], Karolina Sanocka[a], Panagiota Petrou[b], Andrzej Budkowski[a]

[a]M. Smoluchowski Institute of Physics, Jagiellonian University, Łojasiewicza 11,

30-348 Kraków, Poland

[b]Institute of Nuclear & Radiological Sciences & Technology, Energy & Safety, NCSR Demokritos, P. Grigoriou & Neapoleos St, Aghia Paraskevi 15341, Athens, Greece

* Corresponding author

E-mail address: katarzyna.gajos@uj.edu.pl

Section S1. Representative WLRS sensor responses for the anti-STR IgG/STR capture assay and non-specific adsorption examination

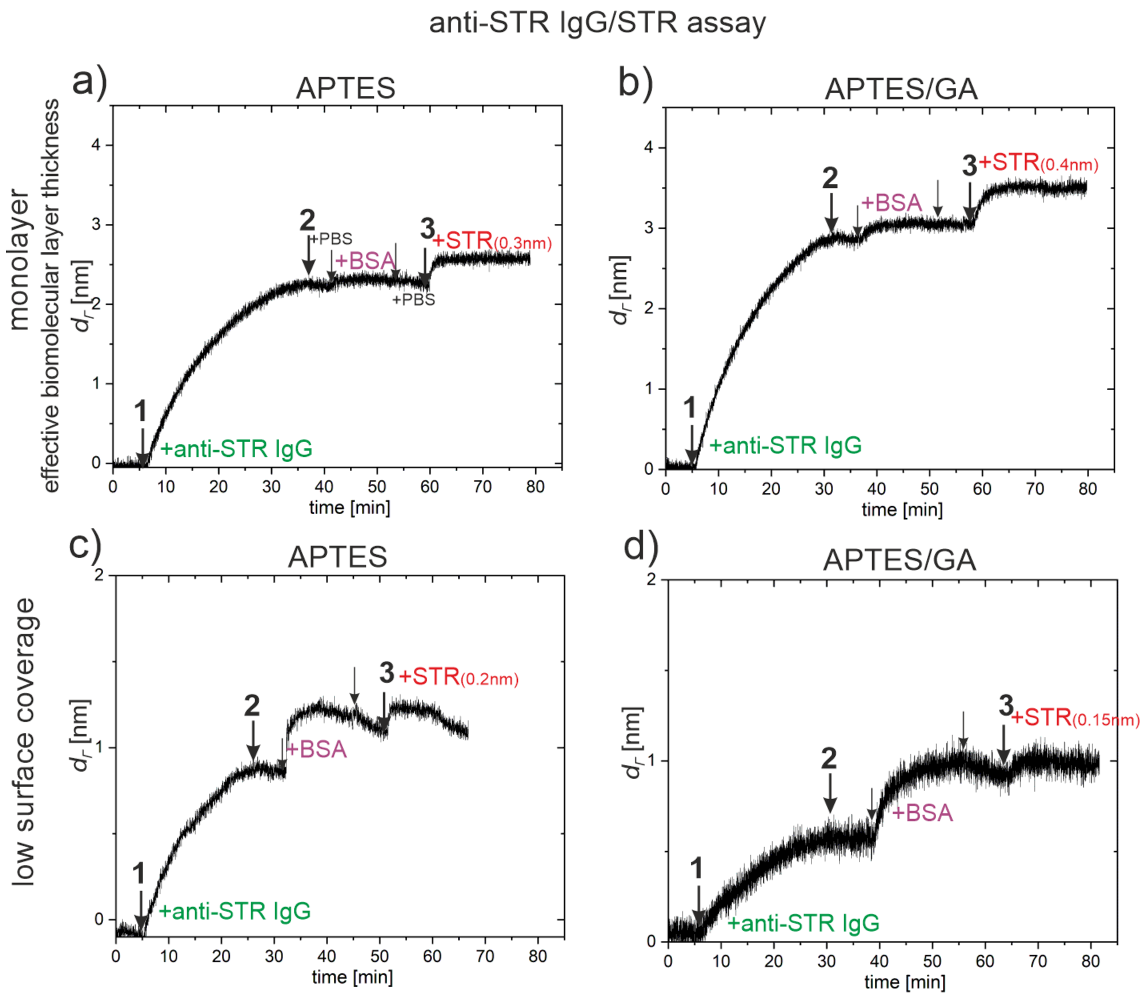

Figure S1. Effective thickness of the biomolecular layer d_r monitored in real time with the WLRS-based biosensor for the anti-STR IgG/STR capture assay carried out on silicon chips modified with APTES **(a, c)** or APTES/GA **(b, d)**, respectively. The results presented were obtained using different concentrations of anti-STR IgG solutions during the immobilization step (1), resulting in the formation of a complete monolayer of IgG molecules (**a, b**; 75 μg/mL) or low surface coverage with IgG molecules (**c**; 20 μg/mL**, d**; 10 μg/mL). The arrows indicate the start of each one of the subsequent protocol steps and mark the solutions run over the biochip.

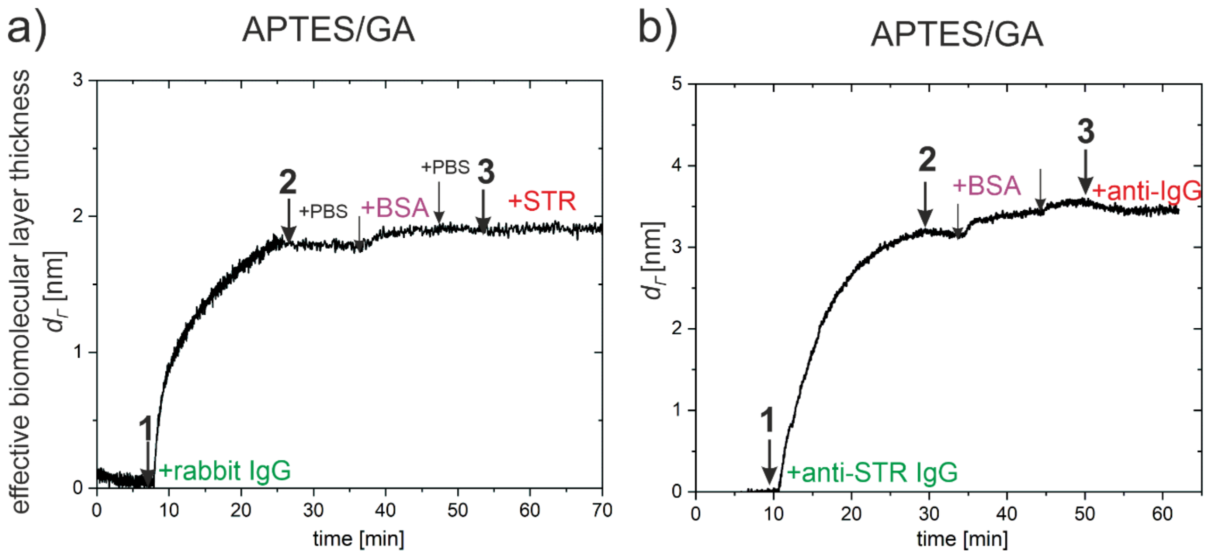

Figure S2. Examination of the non-specific adsorption during assay carried out on silicon chips modified with APTES/GA. Effective thickness of the biomolecular layer d_r monitored in real time with the WLRS-based biosensor for the streptavidin solution (10 µg/mL) flow over the layer of surface immobilized rabbit IgG (a) and anti-rabbit IgG solution (10 µg/mL) flow over the layer of surface immobilized goat anti-STR IgG. The arrows indicate the start of each one of the subsequent protocol steps and mark the solutions run over the biochip.

Section S2. TOF-SIMS examination of multi-protein surface composition after completion of the capture assay

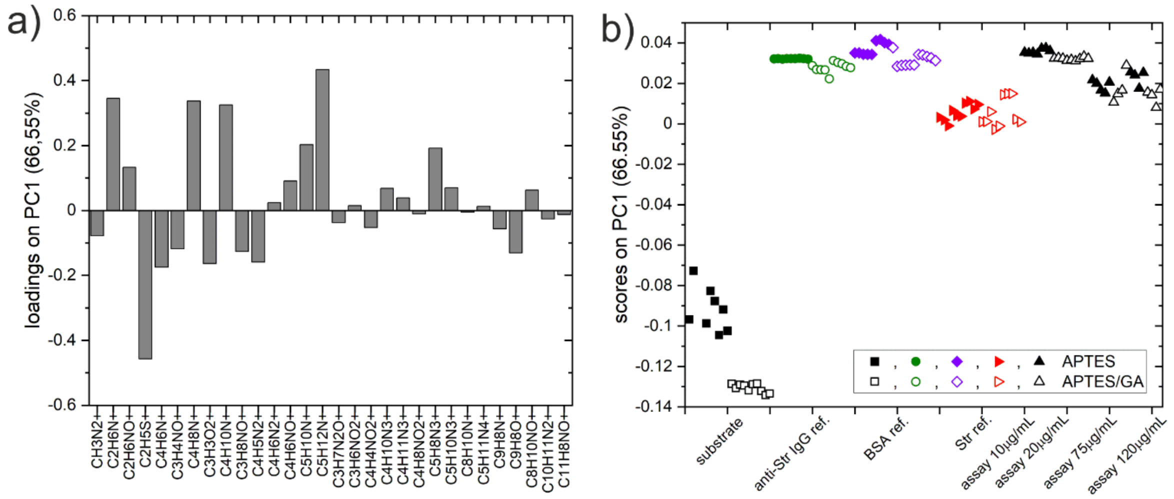

Figure S3. (a) Loadings and **(b)** scores plots for the first principal component in the PCA model presented in Figs. 3 and 4 in the main text.

Section S3. PCA analysis of one-component protein layers with different surface densities

The one-component protein layers of rabbit IgG, STR, and BSA were prepared on APTES modified silicon substrates by applying solutions with different concentrations in the range of 5 µg/mL – 1 mg/mL to obtain samples with different protein surface densities. Protein surface density was determined for each sample by Spectroscopic Ellipsometry. High-resolution positive ion TOF-SIMS spectra were recorded from several (5-10) non-overlapping areas on each sample. PCA analysis involved intensities of amino acid-derived secondary ions normalized to the sum of selected peaks. The PCA model was developed on the basis of data recorded on IgG, STR, and BSA one-component layers with different protein surface density and on a bare APTES surface. The first principal component PC1 orders the data points in terms of the protein surface density for all examined protein layers, as shown in Fig. S4a. In turn, the PC2 and PC3 classifies the data points in terms of the type of protein that forms the layers. As shown in Fig. S4b the point location on the PC3 vs. PC2 scores plot does not depend on the applied solution concentration and hence on the protein surface density.

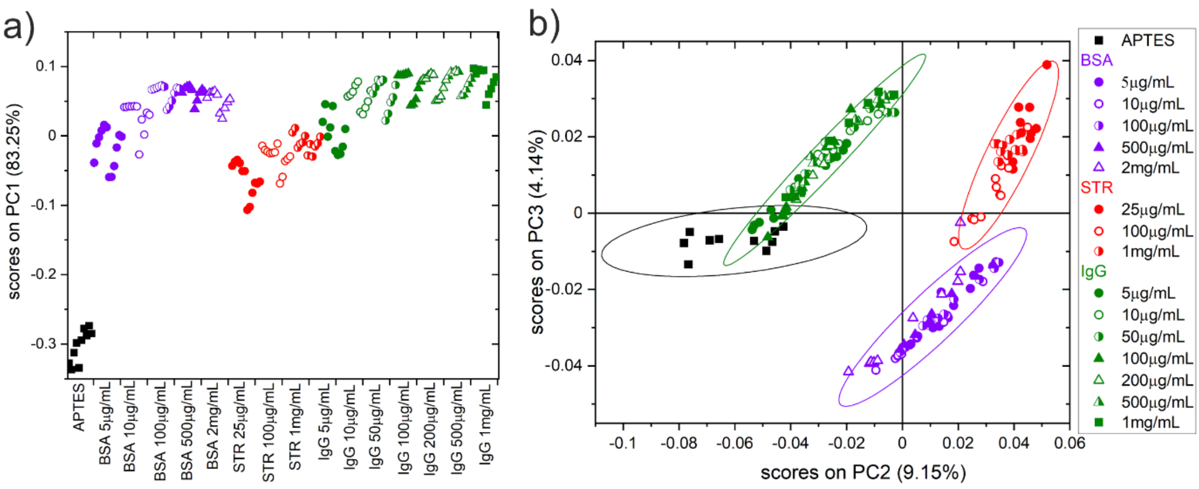

Figure S4. (a) Scores on the first principal component PC1 for the APTES-modified substrate and the layers of BSA, STR and rabbit IgG protein resulted from static immobilization applying solutions with different protein concentrations. (b) PC3 vs. PC2 scores plot for PCA model involving bare APTES modified surface and one-component protein layers of rabbit IgG antibodies, BSA and STR. Data points

are grouped by the type of protein. It is not observed any influence of the concentration of the applied solution on the location of the points.

Section S4. Adsorption isotherms for in-flow immobilization of anti-STR goat IgG

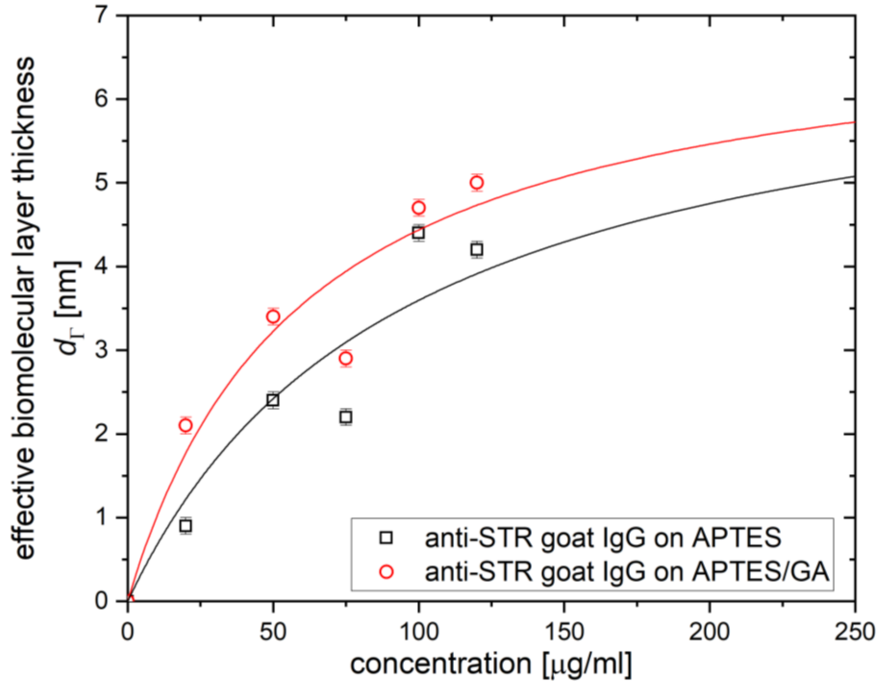

Figure S5. Adsorption isotherms for in-flow immobilization of anti-STR goat IgG on silicon chips modified with APTES (black squares) and glutaraldehyde activated APTES (APTES/GA) (red circles). The effective thickness of the biomolecular layer d_Γ was determined with the WLRS-based biosensor after the immobilization step was completed. The lines in describe the experimental data on the basis of the Langmuir model.

Section S5. Degree of IgG molecules exchanged with BSA determined from the direct assay monitored with WLRS

To evaluate the molecular exchange with BSA of the physisorbed IgG molecules, the following procedure was applied. First, we note that the WLRS signal, recorded for the IgG/anti-IgG (direct) assay on the APTES surface, reflects the thickness d_Γ of the adlayer with all biomolecules (the black line in Fig. S6). This signal is used to determine the molecular binding ratio anti-IgG/IgG, 1.15, as the ratio $d'_{\text{aIgG}}/d'_{\text{IgG}}$ of the WLRS response of the bound anti-IgG (d'_{aIgG}) to that of the surface immobilized IgG (d'_{IgG}). Second, we realize that the *exchange* of the surface-immobilized IgG with BSA during the

blocking procedure is not resolved with WLRS. However, it is absent for the APTES/GA surface, where the respective ratio d_{aIgG}/d_{IgG} is equal to 1.45. Third, for APTES the hypothetical *real* thickness of the surface-immobilized IgG (red line and d_{IgG} in Fig. S6) should be lower than the observed thickness (d'_{IgG}), leading to the *real* increase of d_{aIgG} in the WLRS signal due to the bound anti-IgG. Therefore, the difference between the observed and real WLRS response for the surface-immobilized IgG, d'_{IgG} - d_{IgG}, is equal to the difference between the real and observed response to the bound anti-IgG, $d_{aIgG} - d'_{aIgG}$ (Fig. S6). Thus, using the three relations described above, we obtain the ratio of the real thickness to the observed thickness determined for the surface-immobilized IgG, d_{IgG}/d'_{IgG} ~ 0.88. This points to a 12% level of partial exchange of physisorbed IgG molecules with BSA.

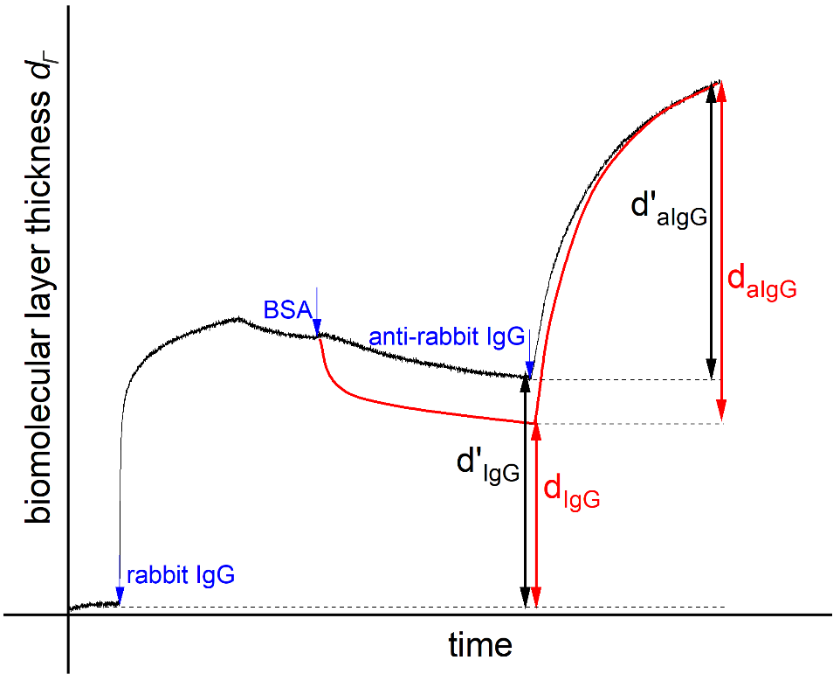

Figure S6. Scheme of a real-time response of the WLRS biosensor recorded for the IgG/anti-IgG (direct) assay on the APTES surface (cf. Figs. 2c). The black line reflects the thickness d_r of the adlayer with all biomolecules used to determine the molecular binding ratio anti-IgG/IgG, 1.15, as the ratio d'_{aIgG}/d'_{IgG} of the WLRS response of the bound anti-IgG (d'_{aIgG}) to that of the surface immobilized IgG (d'_{IgG}). The red line reflects the real thickness of the surface immobilized IgG (d_{IgG}) that leads to the increase in WLRS signal due to the bound anti-IgG (d_{aIgG}). The difference between black and red lines corresponds to the exchange of the surface-immobilized IgG with BSA during the blocking procedure, not resolved with WLRS but absent for the APTES/GA surface, where the ratio d_{aIgG}/d_{IgG} is equal to 1.45.
